# Mutant huntingtin expression in somatostatin-positive interneurons contributes to neurophysiological and behavioral phenotypes in BACHD mice

**DOI:** 10.1242/dmm.052513

**Published:** 2026-03-27

**Authors:** Jahmel A. Fowler, Mariangela Scarduzio, Cayla Pool, Casey D. Mahan, Michelle Gray

**Affiliations:** ^1^ Department of Neurology, Killion Center for Neurodegeneration and Experimental Therapeutics, The University of Alabama at Birmingham Heersink School of Medicine, Birmingham, AL 35294, USA; ^2^ Graduate Biomedical Sciences, Biochemistry and Structural Biology Theme, The University of Alabama at Birmingham Heersink School of Medicine, Birmingham, AL 35294, USA; ^3^ College of Arts and Sciences, Xavier University of Louisiana, New Orleans, LA 70125, USA; ^4^ Summer in Biomedical Sciences (SIBS) Undergraduate Research Program, The University of Alabama at Birmingham, Birmingham, AL 35294, USA

**Keywords:** Huntington's disease, Somatostatin, BACHD, Interneurons, Striatum

## Abstract

Huntington's disease (HD) is caused by expansion of the polyglutamine stretch in the widely expressed huntingtin (HTT) protein. Patients with HD have motor, psychiatric and cognitive changes due to changes in a variety of neural circuits. Somatostatin-expressing interneurons (SST-INs) can regulate neural circuits largely by inhibiting their target cells. Behaviorally, brain-wide inhibition of SST-INs increased anxiety in mice. Silencing striatal SST-INs caused a decrease in movement in the open field. Mutant HTT (mHTT)-expressing mice exhibited abnormal motor, cognitive and psychiatric-like changes, as well as electrophysiological changes in a variety of neurons, including striatal SST-INs. However, it is unknown whether cell-autonomous expression of mHTT in SST-INs contributes to HD-associated behavioral phenotypes or causes abnormal electrophysiological changes in striatal SST-INs. To address these questions, we reduced mHTT expression in SST-INs throughout the brain of BACHD mice. Our findings show that brain-wide reduction of mHTT in SST-INs rescues anxiety-like behavior in male BACHD mice in the light–dark box, without improving performance in the open field or on the rotarod. Additionally, expression of mHTT in striatal SST-INs cell autonomously drives their increased excitability.

## Teaser

**Summary:** Mutant huntingtin expression in somatostatin-expressing interneurons contributes to anxiety-like behavior in male BACHD mice and cell autonomously drives hyperexcitability in these interneurons in dorsolateral striatum.

## INTRODUCTION

Somatostatin-expressing interneurons (SST-INs) are modulators of neural circuits and are known to regulate principal cell activity by synapsing onto the distal dendrites of their target cells (Favuzzi et al., 2019; Gittis and Kreitzer, 2012; Straub et al., 2016). Brain-wide silencing of SST-INs results in increased anxiety in elevated plus maze, open field and phenotyper test paradigms (Fee et al., 2021). In dorsomedial striatum, increasing and silencing SST-IN activity enhances and slows early motor learning on the rotarod, respectively (Rotariu et al., 2025). Silencing SST-INs in dorsolateral striatum reduces movement in an open field paradigm (Qian et al., 2022). In hippocampus and amygdala, SST-IN activity is predictive of anxiolytic behavior in an elevated plus maze paradigm (Jackson et al., 2024). These studies show that SST-INs activity can contribute to many of the behaviors that are altered in Huntington's disease (HD).

HD is a fatal neurodegenerative disorder, inherited in an autosomal dominant pattern (The Huntington's Disease Collaborative Research Group, 1993). Clinically, Patients with HD present with cognitive, psychiatric and movement abnormalities that worsen with disease progression (McColgan and Tabrizi, 2018; Roos, 2010; Ghosh and Tabrizi, 2018). HD is caused by a CAG repeat expansion in exon 1 of the huntingtin (*HTT*) gene, which results in an expanded polyglutamine repeat in the HTT protein (Group, 1993; Orr et al., 1993; Snell et al., 1993; Andrew et al., 1993). The HTT protein is widely expressed in all cells in the brain, including non-neuronal and neuronal cell types (Shin et al., 2005; DiFiglia et al., 1995; Sharp et al., 1995), and it is known to contribute to various cellular functions (Barron et al., 2021; Saudou and Humbert, 2016; Schulte and Littleton, 2011).

Neuropathologically, HD is characterized most prominently by significant striatal atrophy, although other brain regions – including the cerebral cortex, amygdala and hippocampus – are also affected as disease progresses (Matz and Spocter, 2022; Plotkin and Goldberg, 2019; Hedreen and Folstein, 1995; Ahveninen et al., 2018; Wibawa et al., 2023). The striatum, the major input structure of the basal ganglia, is the most affected brain region in HD (Vonsattel et al., 1985; McColgan and Tabrizi, 2018; Bamford and Bamford, 2019). The GABAergic striatal medium spiny neuron (MSN) is the most degenerated cell type in HD (Vonsattel et al., 1985; Richfield et al., 1995; Han et al., 2010; Ferrante et al., 1991). The MSN is the major output cell type of the striatum (Tepper and Bolam, 2004; Matamales et al., 2009). MSNs integrate inputs from multiple brain regions, including excitatory input from thalamic and cortical regions and dopaminergic input from the substantia nigra (Bolam et al., 2000; Gerfen, 1988; Gerfen and Bolam, 2016; Smith et al., 1998; Freeze et al., 2013). They are also modulated by a network of different striatal interneurons, including SST-INs, to coordinate basal ganglia functions (Gittis and Kreitzer, 2012; Bolam et al., 2000; Tepper and Koós, 2016; Tepper et al., 2018, 2004, 2010).

Striatal SST-INs account for less than 1% of striatal neurons, and they synapse on dendrites of their target cells, including projection neurons and other interneurons, contributing to the complex network of inhibitory circuits (Tepper et al., 2010; Figueredo-Cardenas et al., 1996; West et al., 1996). Immunohistochemical studies using both post-mortem tissue from patients with HD and tissue from mutant HTT (mHTT)-expressing mice revealed that striatal SST^+^ interneurons are spared in number and do not have overt morphological changes (Rallapalle et al., 2021; Reiner et al., 2013; Voelkl et al., 2022). Although prior work has suggested that mHTT protein expression in striatal SST-INs is relatively low compared to that in MSNs (Kosinski et al., 1997, 1999), studies assessing cell type RNA expression profiles using single-cell RNA-sequencing experiments show that striatal SST-INs express *HTT* mRNA at levels that are similar to those in MSNs (Saunders et al., 2018) (see Fig. S2). Therefore, the expression of mHTT in these cells may alter their function and, thus, contribute to circuit dysfunction through mechanisms such as disinhibition or maladaptive firing. Studies in mHTT-expressing R6/2, Q175 and BACHD mice revealed increased rates of spontaneous firing of SST-INs (Cepeda et al., 2013; Holley et al., 2019b). In addition, activating striatal SST-INs using channelrhodopsin revealed an increase in inhibitory synaptic input onto MSNs in the R6/2 and Q175 mouse model compared to wild type (WT) (Cepeda et al., 2013; Holley et al., 2019a), whereas optogenetically silencing striatal SST-INs in Q175 mice resulted in a reduction of the observed increase in inhibitory synaptic input onto MSNs (Holley et al., 2019a), suggesting that pathologically increased SST-IN discharge is a likely source of increased MSN inhibition (Cepeda et al., 2013; Holley et al., 2019a). Overall, the evidence pointing to the critical function of SST-INs in motor and anxiety behaviors suggests that mHTT expression in these cells could cause these cells to contribute to the abnormal motor and non-motor behavioral manifestations of HD. Thus, decreasing mHTT expression in SST-INs could improve these behavioral deficits. Similarly, mHTT expression in SST-INs may improve the altered electrophysiological profiles observed in these cells in the striatum.

In this study, we used the conditional full-length human mHTT-overexpressing BACHD mouse model, which recapitulates many of the phenotypes observed in patients with HD, including decreased brain weight and striatal atrophy at >1 year of age (Gray et al., 2008; Wood et al., 2019; King et al., 2020) and progressive motor, cognitive and psychiatric-like behavioral abnormalities (Gray et al., 2008; Wood et al., 2019; King et al., 2020; Molero et al., 2016; Wang et al., 2014). To determine whether brain-wide reduction of mHTT in the SST-INs affected the behavioral phenotypes of the BACHD mice, we genetically reduced mHTT expression globally in SST-INs by breeding BACHD mice to SST-Cre mice. Additionally, given the known alterations of striatal SST-IN function in mHTT-expressing mice, we assessed whether decreasing mHTT in these interneurons would affect the observed electrophysiological deficits. We report that abnormal motor phenotypes observed in BACHD mice on the rotarod and in open field did not improve after reducing mHTT in SST-INs in BACHD mice. However, reduction of mHTT levels in SST-INs of male BACHD mice resulted in amelioration of anxiety-like behavior in the light–dark box paradigm. In addition, we saw a reduction in the previously observed increase in SST-IN spontaneous firing in dorsolateral striatum when we reduced mHTT expression in SST-INs of BACHD mice. This study highlights the complex role of mHTT expressed in different cell types in mediating HD phenotypic manifestations and underscores the need for future studies investigating cell type-specific and region-specific (e.g. using viral SST-Cre expression in specific regions) contributions to HD phenotypes.

## RESULTS

### Mutant huntingtin reduction in SST-INs in BACHD/SST-Cre mice

To understand whether the observed behavioral phenotypes and the electrophysiological changes seen in SST-INs in BACHD mice was due to cell-autonomous expression of mHTT in SST-Ins, we used SST-Cre mice to selectively reduce mHTT levels.

**Fig. 1.**
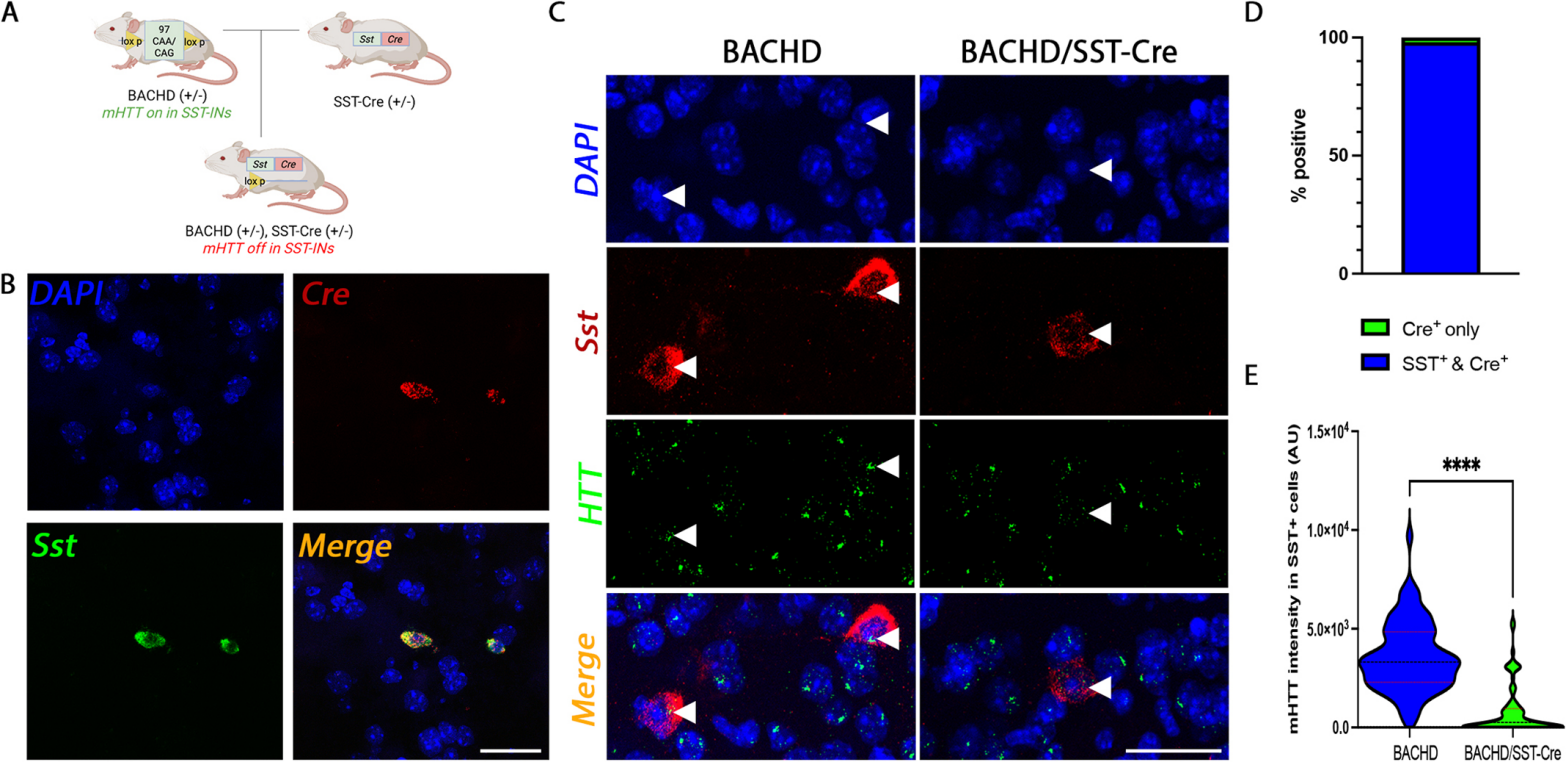
**Cre is selective for striatal somatostatin-expressing interneurons (SST-INs) and reduces mutant huntingtin expression in SST-INs of BACHD mice.** (A) Breeding schematic showing hemizygous BACHD mice bred to heterozygous SST-Cre mice to create double mutant BACHD/SST-Cre mice. Created in BioRender by Fowler, J. A. (2026). https://BioRender.com/s5jqo5z. This figure was sublicensed under CC-BY 4.0 terms. (B) Representative confocal images of Cre selectivity in SST-Cre/wild-type (WT) mice. RNAscope was used to verify Cre selectivity using probes specific to mouse somatostatin (*Sst*) and Cre recombinase (Cre); *n*=3 mice. (C) Representative confocal images of mutant huntingtin reduction in striatal SST-INs of BACHD mice. RNAscope probes specific to mouse *Sst* and human huntingtin (*HTT*) were used; *n*=3 mice. Arrowheads indicate somatostatin-expressing interneurons in BACHD and BACHD/SST-Cre mice. (D) Quantitation of Cre expression in SST-INs cells shows that ∼98% of Cre^+^ cells are also *Sst*^+^. (E) Quantitation of mutant huntingtin (mHTT) reduction in SST-INs of BACHD/SST-Cre mice shows ∼78% reduction in mHTT expression in SST-INs (*P*<0.0001). Mann–Whitney *U*-test, ****P<0.0001. AU, arbitrary units. Scale bars: 50 µm.

The BACHD mouse contains a conditional full-length mutant human *HTT* gene (Gray et al., 2008; Wang et al., 2014). We crossed BACHD mice to Ssttm^2.1(cre)Zjh/^J (SST-Cre) (Taniguchi et al., 2011) mice to reduce mutant expression in SST-INs (Fig. 1A). To validate Cre selectivity, we performed RNAscope fluorescence *in situ* hybridization (FISH) on fresh frozen striatal tissue in 2-month-old SST-Cre/WT mice (Fig. 1B). We used probes specific to mouse somatostatin (*Sst*) and Cre recombinase (Cre). We showed that nearly 98% of Cre^+^ cells in the striatum were also positive for *Sst*, suggesting that Cre recombinase expression is selective for SST-INs (Fig. 1D, *n=*3 mice, 102 cells) in the SST-Cre mice.

To evaluate the effectiveness of mHTT knockdown, we performed FISH experiments on fresh frozen tissue of BACHD and BACHD/SST-Cre mice using probes specific to mouse *Sst* and human *HTT*. In the striatum, we found ∼78% reduction of m*HTT* in striatal SST-INs (*P*<0.0001) (Fig. 1C,E, *n=*3 BACHD mice, 76 cells and *n*=3 BACHD/SST-Cre mice, 80 cells). Taken together, these results showed that we could successfully target SST-INs in these mice and reduce m*HTT* expression in SST-INs.

### Motor coordination is abnormal in BACHD mice and does not improve in BACHD/SST-Cre mice

**Fig. 2.**
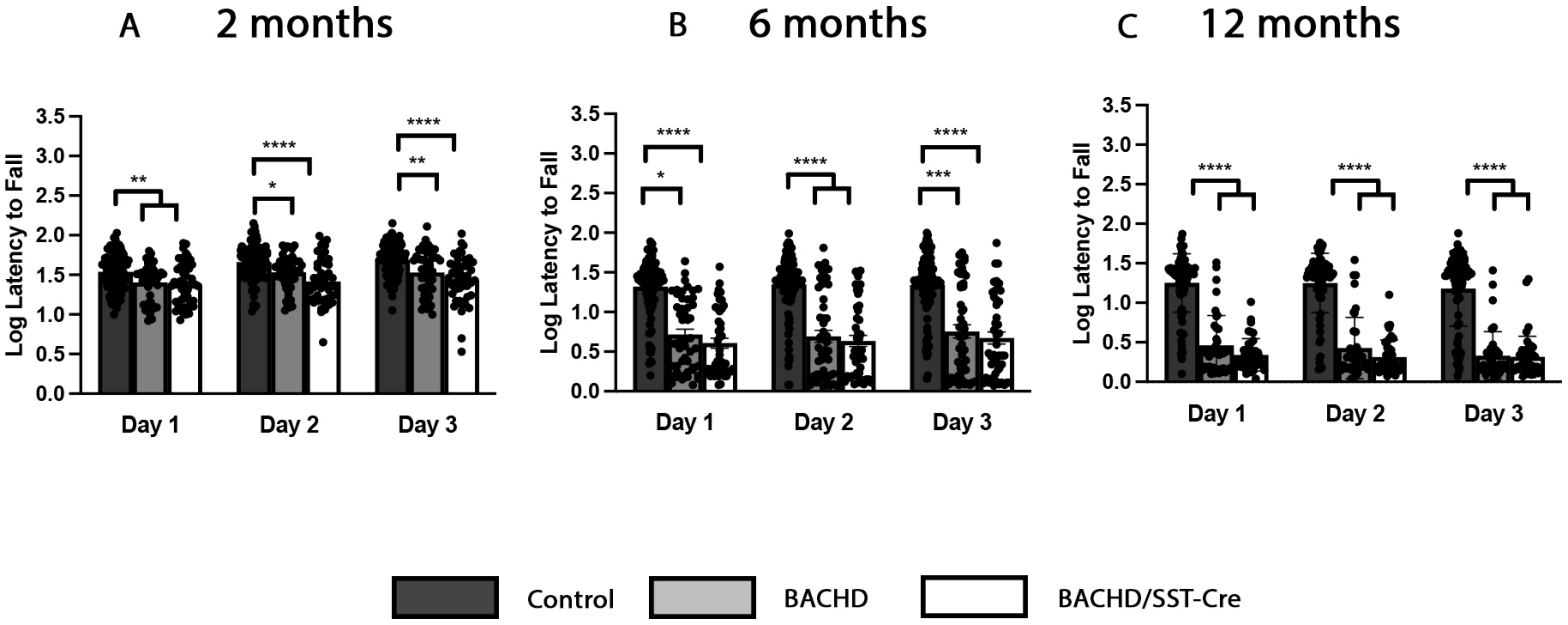
**Reducing mutant huntingtin in SST-INs does not improve motor coordination in BACHD mice.** Rotarod analysis of control, BACHD and BACHD/SST-Cre mice at 2, 6 and 12 months of age. (A) Latency to fall is decreased in BACHD and BACHD/SST-Cre mice compared to control mice at 2 months of age, with no statistically significant difference between the groups. (B,C) This is also seen at 6 months (B) and 12 months (C). Data were analyzed using a two-way repeated measure ANCOVA and adjusted for weight and genotype interactions. Tukey's HSD was used for post hoc comparisons. 2 months, *n*=44-105/genotype; 6 months, *n*=49-100/genotype; 12 months, *n*=41-83/genotype. Sample sizes by sex and genotype are available in Table S3. *P<0.05, **P<0.01, ***P<0.001, ****P<0.0001.

**Fig. 3.**
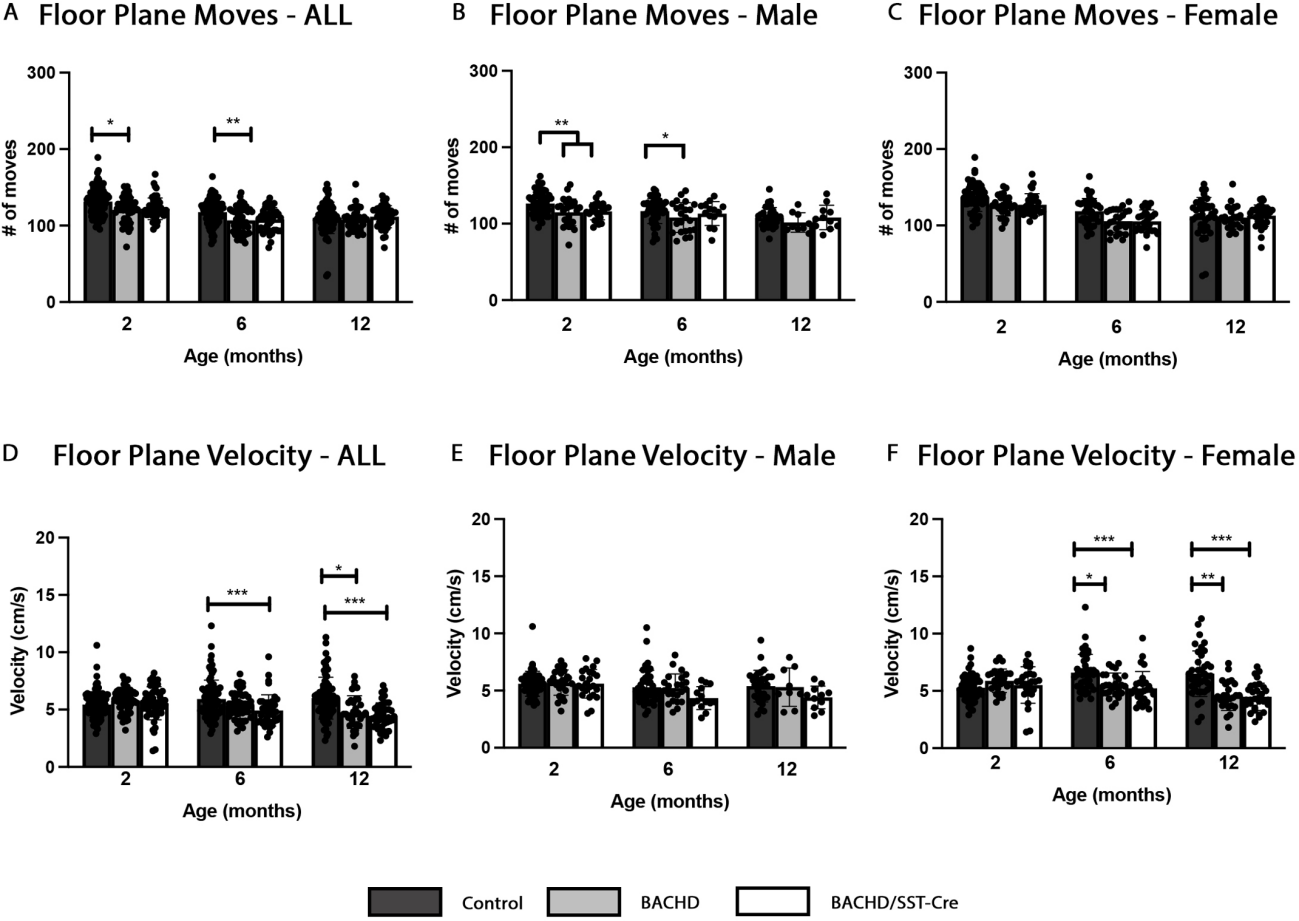
**Motor abnormalities are not improved in BACHD/SST-Cre mice.** Open field analysis of control, BACHD and BACHD/SST-Cre mice at 2, 6 and 12 months of age. (A) Floor plane moves are decreased in the whole cohort of BACHD mice at 2 months (*P*=0.0216) and 6 months (*P*=0.0079) compared to control mice. (B) Floor plane moves are decreased in male BACHD (*P*=0.0019) and BACHD/SST-Cre mice (*P*=0.0083) at 2 months of age relative to those in control mice. At 6 months of age, when mice are separated by sex, only male BACHD mice have a significant decrease in floor plane moves (*P*=0.0374) compared to control mice. (C) No significant differences in floor plane moves were observed among female mice. (D) As a cohort, floor plane velocity is decreased in BACHD/SST-Cre mice at 6 months of age (*P*=0.0006) relative to that in control mice. At 12 months of age, both BACHD (*P*=0.0123) and BACHD/SST-Cre (*P*=0.0008) show decreased velocity relative to that in control mice. (E) No significant differences were observed in floor place velocity among male mice. (F) Female BACHD (6 months, *P*=0.0110; 12 months, *P*=0.0019) and BACHD/SST-Cre (6 months, *P*=0.0003; 12 months, *P*=0.0005) mice show decreased floor plane velocity at 6 and 12 months of age compared to control mice. A two-way repeated measure ANCOVA, adjusting for weight and genotype interaction, was used to analyze the data. Tukey's HSD was used for post hoc comparisons. 2 months, *n*=50-107/genotype; 6 months, *n*=41-87/genotype; 12 months, *n*=42-71/genotype. Sample sizes by sex and genotype are available in Table S3. *P<0.05, **P<0.01, ***P<0.001.

Motor coordination deficits, as determined by using an accelerated rotarod paradigm, are observed in BACHD mice as early as 2 months of age and get progressively worse as the animal ages, with more significant decline observed at 12 months of age (Wood et al., 2019; Wang et al., 2014). To assess whether dysfunctional SST-INs contribute to the loss of motor coordination seen in BACHD mice, we tested control (WT/WT and SST-Cre/WT mice combined), BACHD and BACHD/SST-Cre mice on the accelerating rotarod using a repeated measure paradigm. Owing to the progressive nature of BACHD mice, any improvement in deficits may not be seen until 12 months of age (Wood et al., 2019). Thus, we performed rotarod experiments at 2, 6 and 12 months of age. Because body weight differs across genotypes (Table S2) and may influence rotarod performance, we analyzed the data using a two-way repeated measure ANCOVA, which included weight as a covariate and genotype as a fixed factor, along with their interaction. We adjusted for the interaction between weight and genotype in our model. There was a significant effect of genotype at 2 [*F*(2,124)=11.54, *P*=2.54×10^−5^], 6 [*F*(2,124)=49.40, *P*=1.66×10^−16^] and 12 [*F* (2,124)=21.47, *P*=9.48×10^−28^] months of age. There was not an effect of weight at 2 months of age [*F*(1,125)=7.17×10^−3^, *P*=0.933], but there was a significant effect of weight at 6 [*F*(1,125)=2.33, *P*=7.96×10^−4^] and 12 [*F*(1,125)=3.86, *P*=0.933, *P*=1.01×10^−7^] months. We saw a decline in motor coordination beginning at 2 months of age, which progressed through 12 months of age in BACHD mice (Fig. 2). BACHD/SST-Cre mice also showed a decline in motor performance beginning at 2 months of age, which also became more pronounced at 12 months (Fig. 3). There was no improvement in motor coordination in BACHD and BACHD/SST-Cre mice at 2 (Day 1, *P*=0.8007; Day 2, *P*=0.1194; Day 3, *P*=0.3617), 6 (Day 1, *P*=0.5745; Day 2, *P*=0.8901; Day 3, *P*=0.4907) or 12 (Day 1, *P*=0.8357; Day 2, *P*=0.9576; Day 3, *P*=0.9240) months of age (Fig. 2A-C). To assess whether there was any contribution to performance based on sex of the mice, we examined male and female mice separately. The performances of both male and female BACHD mice declined with age, and BACHD/SST-Cre male and female mice did not differ from BACHD mice (Fig. S1). These data suggest that there was no effect of reducing mHTT expression in SST-INs on the gross motor coordination deficit in the accelerated rotarod paradigm in BACHD mice.

### Deficits in locomotor activity in BACHD mice in the open field do not improve in BACHD/SST-Cre mice

Locomotor activity in the open field is reduced in BACHD mice (Gray et al., 2008; Wood et al., 2019; King et al., 2020). However, it is unknown whether SST-INs contribute to this phenotype. To evaluate the contribution of mHTT expressing SST-INs to general locomotor activity, we used a repeated measure open field paradigm to test control (WT/WT and SST-Cre/WT), BACHD and BACHD/SST-Cre mice. Similarly to in our rotarod studies, we analyzed the open field data using a two-way repeated measure ANCOVA, in which we included weight and genotype as a covariate and fixed factor, respectively.

There was a significant effect of genotype [*F*(2, 237)=12.66, *P*=5.979752×10^−6^] and weight [*F*(1,237)=2.60, *P*=6.42×10^−7^] on floor plane moves in the open field arena. After adjusting for weight and genotype interactions, as a cohort, BACHD mice had fewer floor plan moves in the open field arena than did control mice (*P*=0.0216) (Fig. 3A). This trend persisted at 6 months of age, when only BACHD (*P*=0.0079) mice had fewer floor plane moves than control mice (Fig. 3A), indicating an age-dependent reduction in generalized locomotor activity in BACHD mice. When data were segregated by sex, male BACHD (*P*=0.0019) and BACHD/SST-Cre (*P*=0.083) mice had fewer floor plane moves than control mice at 2 months of age, while at 6 months, only male BACHD mice had fewer floor plane moves relative to those of control mice (*P*=0.0374) (Fig. 3B), with a significant effect of genotype [*F*(2,110)=4.96, *P*=8.681015×10^−3^]. When only female mice were analyzed at 6 months, BACHD (*P*=0.0581) and BACHD/SST-Cre (*P*=0.0637) mice had fewer floor plane moves than control mice. However, these differences were not statistically significant (Fig. 3C).

In addition, there was a significant effect of both weight [*F*(1,236)=1.493, *P*=2.99×10^−2^] and genotype [*F*(2,237)=6.63, *P*=1.580×10^−3^] on floor plane velocity. When adjusting for weight and genotype interactions, we did not see any significant differences between genotypes when examining floor plane velocity at 2 months of age as a cohort (Fig. 3D). At 6 months of age, BACHD/SST-Cre (*P*=0.0006) mice showed a significant decrease in floor plane velocity relative to that of control mice (Fig. 3F) as a cohort. At 12 months of age, both BACHD (*P*=0.0123) and BACHD/SST-Cre (*P*=0.0008) mice had significantly lower floor plane velocity than control mice (Fig. 3D) as a cohort. When data were segregated by sex, at 6 months of age, female BACHD (*P*=0.0110) and BACHD/SST-Cre mice (*P*=0.0003) displayed a decline in floor plane velocity compared to that of control mice (Fig. 3F). Also, at 12 months of age, female BACHD (*P*=0.0019) and BACHD/SST-Cre (*P*=0.0005) mice had decreased floor plane velocity compared to that of control mice. There were no significant differences seen in male mice at any of the time points analyzed (Fig. 3E).

### Male BACHD mice display an anxiety-like behavior that is ameliorated when mHTT is reduced in SST-INs

**Fig. 4.**
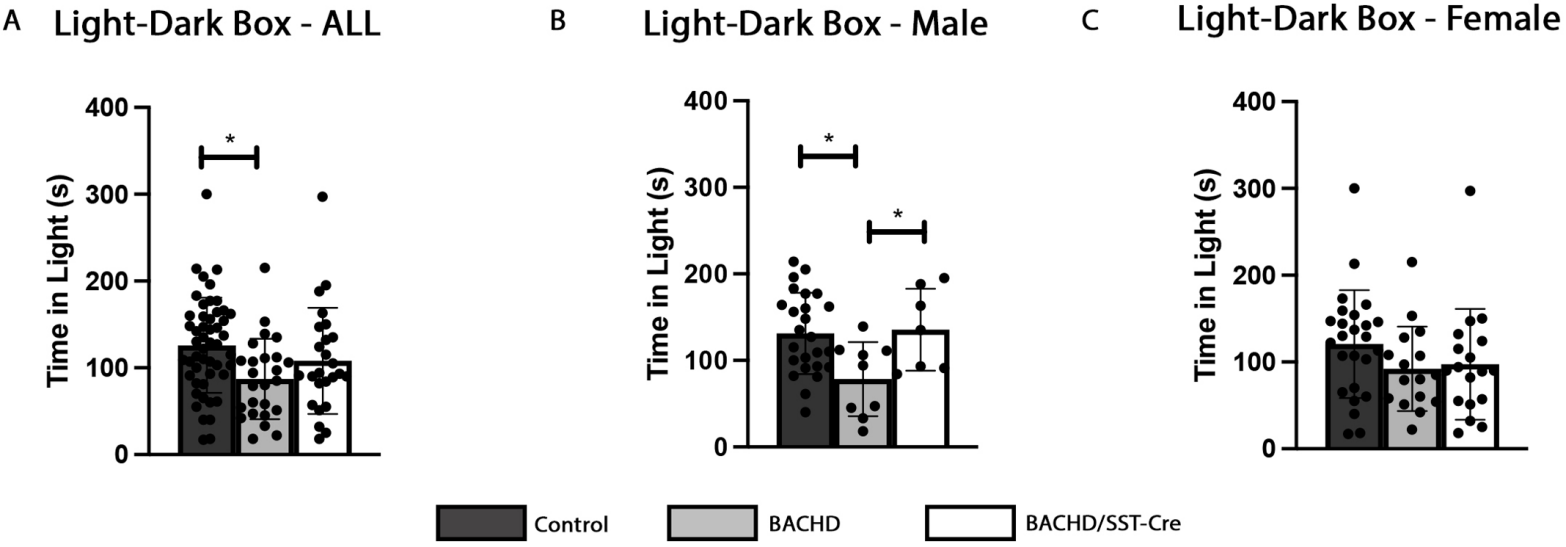
**Anxiety-like behavior is rescued in male BACHD/SST-Cre mice.** Light–dark box analysis of aged control, BACHD and BACHD/SST-Cre mice. (A) In the entire cohort, there is no improvement in time spent in the light in BACHD/SST-Cre mice compared to control mice (*P*=0.3778). (B) Male BACHD/SST-Cre mice spend significantly more time in the light than BACHD mice (*P*=0.0148), with no difference observed between male control and male BACHD/SST-Cre mice (*P*=0.9739), suggesting a rescue in anxiety-like behavior. (C) Female mice show no significant difference among all genotypes. A one-way ANOVA was used. Tukey's HSD was used for post hoc comparisons; *n*=25-50/genotype. Sample sizes by sex and genotype are available in Table S3. *P<0.05.

BACHD mice have increased anxiety in the light–dark box paradigm (Wood et al., 2019). We tested whether reducing mHTT expression in SST-INs would decrease the anxiety-like phenotype in BACHD/SST-Cre mice in the light–dark box. In this paradigm, there was no effect of weight [*F*(2,97)=2.229, *P*=0.139] as a cohort. However, we saw a significant effect of genotype on time spent in the light [*F* (2,97)=4.29, *P*=0.0164] in this cohort of mice. BACHD mice spent significantly less time in the light than control mice (*P*=0.0126), with no differences seen when control mice were compared to BACHD/SST-Cre mice (*P*=0.3682) (Fig. 4A).

When separated by sex, there was a significant effect of genotype on the performance of male mice in the light–dark box paradigm [*F*(2,38)=4.79, *P*=0.0139], with no significant effect of weight [*F*(2,38)=4.79, *P*=0.165]. Male BACHD mice spent significantly less time in the light than control mice (*P*=0.0148) (Fig. 4B). Interestingly, male BACHD/SST-Cre mice and male control mice spent a similar amount of time in the light (*P*=0.9739) (Fig. 4B). In female mice, however, no significant effect of genotype [*F*(2,56) =1.39, *P*=0.2581] or weight [*F*(2,56)=1.39, *P*=0.412] was observed (Fig. 4C). These results suggested that reduction of mHTT expression in SST-INs improves anxiety-like phenotypes in male BACHD mice.

### Reducing mHTT in striatal SST-INs normalizes their spontaneous firing rate

**Fig. 5.**
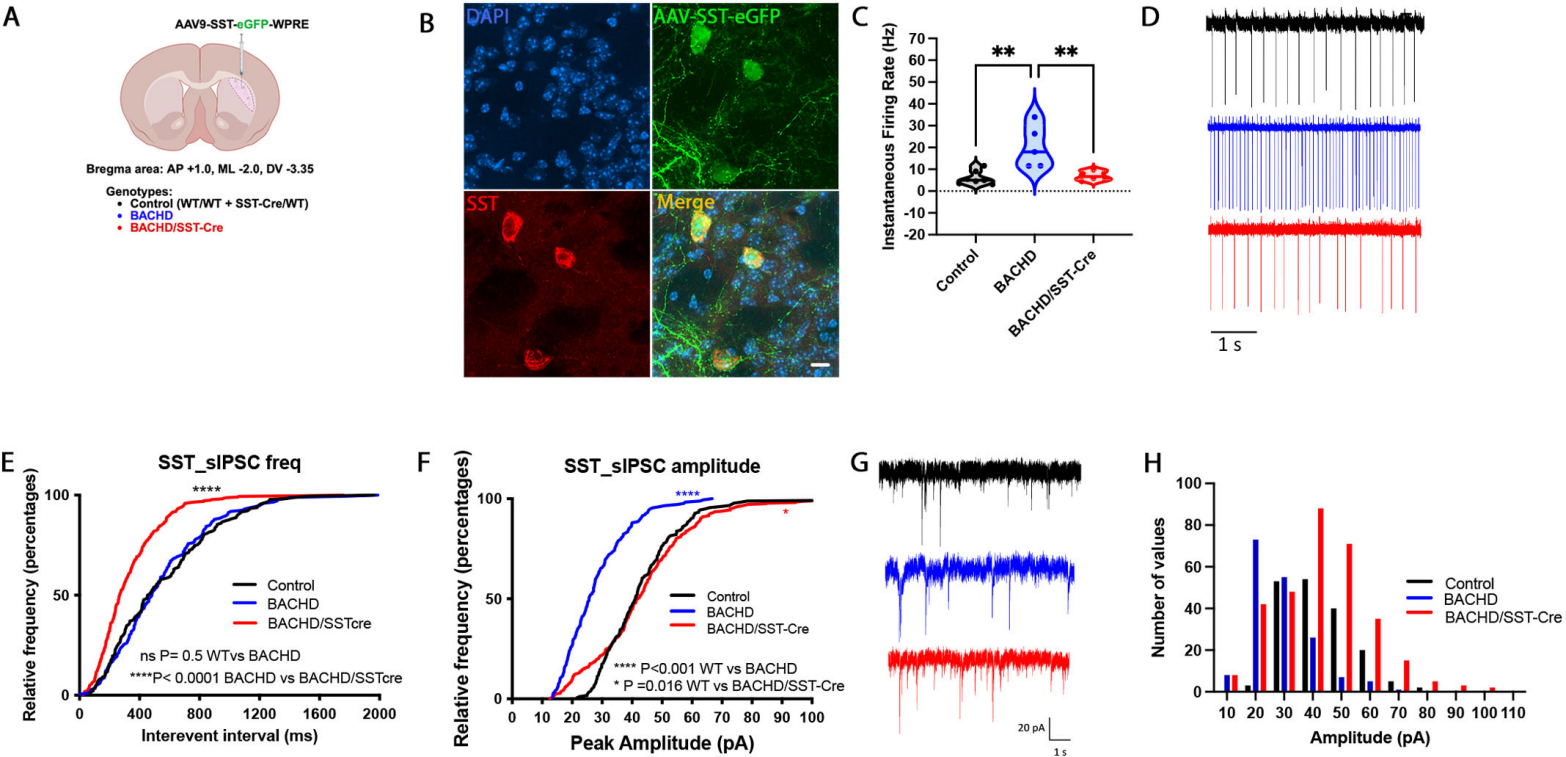
**Striatal SST-INs show decreased spontaneous firing rate and increased inhibitory inputs after reduction of mHTT in SST-INs.** (A) Schematic showing the location of the AAV9-SST-eGFP injection site in control (WT/WT+SST-Cre/WT mice), BACHD and BACHD/SST-Cre mice. Created in BioRender by Fowler, J. A. (2026). https://BioRender.com/g1rmotx. This figure was sublicensed under CC-BY 4.0 terms. (B) Representative confocal images of striatal tissue injected with AAV9-SST-eGFP-WPRE (green) and stained using a SST-specific antibody (red). Scale bar: 10 µm. (C) Instantaneous firing rate of SST-INs is increased in BACHD mice compared to that in control mice (BACHD, *N*=3, *n*=10; control, *N*=4, *n*=17) (*P*=0.0015) but not significantly different between control and BACHD/SST-Cre mice (*N*=6, *n*=38) (*P*=0.9468). Single points represent *N*; lines represent median values. (D) Color-coded spontaneous firing sample traces. (E) Cumulative distribution of inter-event intervals of spontaneous inhibitory and excitatory postsynaptic currents (sIPSCs), showing that the frequency of events is not different between SST-INs from control (*N*=7, *n*=8) and BACHD mice (*N*= 5, *n*=6) (*P*=0.5) but is significantly increased in SST-INs from BACHD/SST-Cre mice (*N*=6, *n*=9) (*P*<0.0001). (F) Cumulative distribution of sIPSC amplitude shows significantly reduced events in SST-INs from BACHD mice relative to those in SST-INs from control mice (*P*<0.0001), which is normalized in the BACHD/SST-Cre mice (*P*=0.016). (G) Color-coded sIPSC sample traces. (H) Color-coded sIPSC amplitude-frequency histogram, showing the distribution of amplitude events in control, BACHD and BACHD/SST-Cre mice. *N* denotes the number of mice; *n* denotes the number of cells. Analysis of spontaneous firing was performed using a one-way ANOVA, with Tukey's HSD for post hoc comparisons. sIPSC amplitude and frequency was analyzed using Kolmogorov–Smirnov test. Sample sizes by sex and genotype are available in Table S4. *P<0.05, **P<0.01, ****P<0.0001.

Multiple mHTT-expressing mouse models display increased spontaneous firing of striatal SST-INs (Cepeda et al., 2013; Holley et al., 2019a). Whether mHTT expression in these cells contributes to this increase is unknown. To answer this question, we unilaterally injected control (WT/WT+SST-Cre/WT), BACHD and BACHD/SST-Cre mice with AAV9-SST-eGFP-WPRE to label SST-INs for patch-clamp recordings (Fig. 5A). Patch-clamp cell-attached recordings of eGFP^+^ cells that were spontaneously active revealed the previously described increase in spontaneous firing of SST-INs in BACHD mice relative to that in control littermates (Cepeda et al., 2013) (Fig. 5B,C). Interestingly, in BACHD/SST-Cre mice, the spontaneous firing rate of SST-INs was not significantly different from that in the control group (*P*=0.9468) (Fig. 5B,C), suggesting that reducing mHTT expression in striatal SST-INs is sufficient to normalize their intrinsic activity in a cell-autonomous way. Synaptic inputs have the potential to modulate SST-IN activity. Previous data showed that the frequency of inhibitory inputs onto SST-INs is decreased in BACHD mice (Cepeda et al., 2013). In this study, we could not replicate this finding in this cohort of mice. In fact, spontaneous inhibitory and excitatory postsynaptic currents (sIPSCs) recorded from SST-INs from control (WT/WT+SST-Cre/WT) and BACHD mice showed no difference in frequency (Fig. 5D,F), but the amplitude of the inhibitory events was reduced in BACHD mice compared to that in control mice (Fig. 5E-G). Interestingly, both the frequency and amplitude of sIPSCs were significantly elevated in SST-INs from BACHD/SST-Cre mice relative to those from control and BACHD mice (Fig. 5D-G), indicating increased inhibitory synaptic inputs to the interneurons after mHTT reduction.

## DISCUSSION

SST-INs are important contributors to neural circuits throughout the brain. These cells release both GABA and SST to inhibit output neuron activity (Liguz-Lecznar et al., 2022; Pittaluga et al., 2021). SST-INs can modulate anxiety and motor phenotypes in mice (Liguz-Lecznar et al., 2022; Pittaluga et al., 2021). As described above, brain-wide chemogenetic silencing of SST-INs increased anxiety in mice (Fee et al., 2021). Interestingly, optogenetic silencing of striatal SST-INs caused the mice to move less total distance in an open field (Qian et al., 2022). However, it was unknown whether mHTT-expression in SST-INs contributed to motor and psychiatric deficits observed in HD.

We analyzed the contribution of mHTT-expressing SST-INs to behavioral phenotypes by globally reducing mHTT in SST-INs of BACHD mice. Previous characterization of the behavioral phenotypes of the BACHD mouse model revealed changes in motor function, with progressive decreases in overall locomotor activity and motor coordination with age in the open field (general locomotor activity) and on the rotarod (motor coordination). The results of the present study largely align with previous behavioral results obtained using the BACHD model (Wood et al., 2019; King et al., 2020; Wang et al., 2014). We did not see any improvement in motor deficits in BACHD/SST-Cre mice compared to BACHD mice, indicating that reduction of mHTT in SST-INs was not sufficient to rescue the types of motor deficits detectable with open field or rotarod tests. We acknowledge that BACHD mice have increased body weight, relative to that of control mice, and this may influence the ability of the mice to perform behavioral tasks. As a result, open field and rotarod experiments were adjusted for weight and genotype interactions, and we still observed differences in the performances of these mice in these tasks.

Patients with HD show many types of motor dysfunction, including chorea, abnormal gait, bradykinesia and akinesia. In HD, choreiform movements typically appear first and are associated with a hyperkinetic (more generalized movements) phenotype. The motor abnormalities can transition to dystonic and bradykinetic movements and, ultimately, akinesia – although choreiform and bradykinetic abnormalities may coexist (Goh et al., 2018; Berardelli et al., 1999). In BACHD mice, we mainly observe what could be described as a hypokinetic phenotype (Gray et al., 2008; Wang et al., 2014; Andre et al., 2011; Menalled et al., 2009). Although the open field test can reveal altered locomotor activity (included in ambulatory distance moved and time moving), this test is not likely to reveal finer motor changes that could be caused by the presence of mHTT in mouse models. Furthermore, chorea-like phenotypes (usually described in mice as rapid, abrupt or irregular jerking) are not often seen in mHTT-expressing mice (Shenoy et al., 2022) and are not likely to be captured by examining distance moved in an open field arena. Importantly, SST-INs serve to modulate principal or output neurons in a circuit, such as MSNs in the striatum (Shenoy et al., 2022). Therefore, these cells may subtly alter the manifestation of motor phenotypes; thus, assessing phenotypes such as stereotypic movements and gait changes may be more sensitive measures of the modulatory roles of these cells in motor circuits. Analyses such as these could be undertaken in future studies because they may be more likely to reveal less overt motor deficits.

The motor circuit is complex and involves multiple cell types in many brain regions. In the striatum, proper MSN function and output requires balance in the activity of the intrastriatal and extrastriatal neurons. MSNs are modulated by multiple types of striatal interneurons, including parvalbumin and cholinergic (Gittis and Kreitzer, 2012; Straub et al., 2016; Qian et al., 2022). Although striatal SST-INs are important for motor function, modulation of mHTT expression only in this cell type may not be sufficient to rescue the abnormal motor phenotypes observed. Owing to the presence of mHTT in all the cells in the striatum, the normal function of these neurons may have been altered to compensate for the presence of mHTT within other neuronal cell types in the microcircuit (including the MSNs).

Previous electrophysiological studies pointed to a prominent role for striatal SST-INs in the increase in inhibitory inputs onto MSNs. However, there was a decrease in the density of striatal parvalbumin-expressing interneurons (PV-INs), with remaining PV-INs exhibiting increased inhibition and altered morphology (Rallapalle et al., 2021; Reiner et al., 2013; Holley et al., 2019b). It is possible that increased SST-IN firing is a maladaptive change for the loss and dysfunction of these PV-INs. Although not evaluated here, it is possible that there is a change in the activity of PV-INs in BACHD mice after reducing mHTT in SST-INs to compensate for the decrease in SST-IN firing in the BACHD/SST-Cre mice. Therefore, reducing mHTT expression from only one interneuron population may not be sufficient to rescue the motor phenotypes assessed with the paradigms used in this study. To see an effect on motor phenotypes, one may need to alter expression of mHTT in multiple interneurons or other cell types simultaneously. This has been seen when mHTT was reduced only in MSNs, where there was no improvement in motor coordination on the rotarod or floor plane distance in the open field (Wang et al., 2014). However, reduction of mHTT in both MSNs and cortical projection neurons was necessary to alleviate these deficits in BACHD mice (Wang et al., 2014).

An increase in anxiety has been described clinically in both male and female patients with HD with no sex-specific difference (Anderson et al., 2018). This has been recapitulated in the BACHD mice in previous studies that showed an increase in anxiety-like phenotypes in both male and female BACHD mice in the light–dark box paradigm relative to those of control mice (Wood et al., 2019; King et al., 2020). In the cohort of mice used in this study, we found that female BACHD and female BACHD/SST-Cre mice spent ∼24% (*P*=0.29) and ∼20% (*P*=0.41) less time in the light, respectively, than female control mice; however, this difference never reached statistical significance. In the cohort of mice used in this study, however, only male BACHD mice displayed an anxiety-like phenotype compared to male control mice (*P*=0.0148), which was rescued by reduction of mHTT in SST-INs using the light–dark box paradigm (Fig. 4). We recognize that using one assay to assess the anxiety-like phenotypes can cause interpretative limitations. Further studies should be done to thoroughly assess these phenotypes using multiple tests. Additionally, future studies could assess locomotor activity in the light–dark box paradigm and assess the contribution of movement and weight to performance in this test.

Expression of the HTT protein is found throughout the brain. Our electrophysiological studies have focused on striatal SST-INs owing to their important role in modulating the function of MSNs, the most vulnerable cell type in HD. We recognize that SST-INs are present throughout the brain; therefore, our genetic strategy likely results in a decrease in mHTT expression in SST-INs in many brain regions, including amygdala, prefrontal cortex, hypothalamus and hippocampus, which are all involved in the regulation of anxiety. Recent studies showed that altering SST-IN beta-synchrony in basolateral amygdala and hippocampus bidirectionally alters anxiety behavior (Jackson et al., 2024). It must be noted that in patients with HD, there is a reduction in the volume of the amygdala even before a clinical diagnosis (Ahveninen et al., 2018). Therefore, it is possible that functional changes in SST-INs in other brain regions may contribute to the anxiety-like phenotype in HD. There are data indicating that SST-IN function may differ between males and females owing to modulation by the estrous cycle, as shown by studies in hypothalamus (Estupina et al., 1996; Massa et al., 2023). Studies in the prefrontal cortex show sex-specific transcriptomic changes in SST-INs of male mice when they are exposed to chronic stress (Girgenti et al., 2019). Therefore, our results showing significant changes in anxiety only in males after reducing mHTT in SST-INs seem to reveal a similar contribution of sex to this phenotype, even in the context of HD. Although we could speculate that normalized activity of striatal interneurons could contribute to the rescue of the anxiety behavior found in this study, this can only be confirmed by knocking down mHTT selectively in striatal SST-INs to directly investigate their role in this behavior.

Multiple studies have focused on electrophysiological changes of striatal SST-INs in mHTT-expressing mice, given that the striatum is most affected in HD. These studies demonstrated altered synaptic input onto striatal SST-INs, as well as increased spontaneous firing of striatal SST-INs across both transgenic and knock-in mHTT-expressing mice (Cepeda et al., 2013; Holley et al., 2019b). Optogenetic silencing of striatal SST-INs resulted in decreased inhibitory input onto MSNs, whereas optogenetic activation of striatal SST-INs caused faster inhibition of MSNs in Q175 mice (Holley et al., 2019a). However, it is unknown whether mHTT expression in striatal SST-INs contributes to altered electrophysiological profiles of SST-INs in the dorsolateral striatum of mHTT-expressing mice. We found that reduction of mHTT in striatal SST-INs can reduce the observed increased firing in BACHD mice (Fig. 5C). Although mice used for electrophysiological studies were used in behavioral testing, we were able to recapitulate the observed increase in firing of striatal SST-INs in BACHD mice as observed in other studies using this model and in other mHTT-expressing mice, for which behavioral studies were not conducted (Cepeda et al., 2013; Holley et al., 2019b).

We acknowledge involvement of extrastriatal cell types that may influence striatal activity. For example, in the cortex, SST-INs function to inhibit principal cell activity (Tremblay et al., 2016). As a result, this global knockdown of mHTT in SST-INs can potentially influence cortical input to the striatum. Therefore, as mentioned, we cannot solely attribute the results we obtained to striatal SST-IN function. Region-specific knockdown would help to elucidate striatal SST-IN function and their regional involvement in HD pathogenesis. Overall, this work highlights the complex contribution of mHTT in a specific cell type to the behavioral and electrophysiological changes in HD.

## MATERIALS AND METHODS

### Animals

All experiments were completed in accordance with the University of Alabama at Birmingham (UAB) Institutional Care and Use Committee and the National Institutes of Health Guide for the Care and Use of Laboratory Animals. Male and female heterozygous knock-in Sst^tm2.1(cre)Zjh^/J (SST-Cre/WT) mice (The Jackson Laboratory, Stock No. 013044) (Taniguchi et al., 2011) were backcrossed to the FvB/NJ background (The Jackson Laboratory, Stock No. 001800) as FVB/*N*-Tg(HTT*97Q)IXwy/J (BACHD) mice (The Jackson Laboratory, Stock No. 008197) (Gray et al., 2008). Mice positive for the SST-Cre gene were bred to hemizygous BACHD mice to obtain WT/WT, BACHD, SST-Cre/WT and BACHD/SST-Cre mice.

### Stereotaxic surgery

Mice were stereotaxically injected into the dorsolateral striatum of 12- to 15-month-old WT/WT, SST-Cre/WT, BACHD and BACHD/SST-Cre mice (2 μl, 7.6×10^12^ genome copies/ml, 0.5 μl/min) using the following coordinates relative to bregma: AP +1.0 mm, anterior and ML −2.0 mm, DV −3.35 mm from the dura. AAV-SST9-eGFP-WPRE (Vector Biolabs, VB5371) was injected for patch-clamp recordings from SST-INs. Sections were stained with an anti-SST antibody (BMA Biomedical, T-4103; 1:500) to verify that cells are SST-INs.

### Electrophysiological recordings

A subset of mice from each genotype that had previously undergone behavioral testing were subsequently injected with AAV9-SST-eGFP-WPRE to selectively label SST-INs for *ex vivo* patch-clamp recordings.

#### Slice preparation

Mice were anesthetized with isoflurane and transcardially perfused with ice-cold high sucrose-modified slicing solution containing 85 mM NaCl, 2.5 mM KCl, 1 mM CaCl_2_, 4 mM MgCl_2_, 1 mM NaH_2_PO_4_, 25 mM NaHCO_3_, 25 mM glucose, 75 mM sucrose (all from Sigma-Aldrich). The whole brain was removed, and corticostriatal sagittal slices (280 μm) were prepared in ice-cold slicing solution using a Compresstome (Precisionary, VF-310-0Z). Slices were then recovered for 20-30 min at 32°C in artificial CSF (aCSF) containing 124 mM NaCl, 4.5 mM KCl, 1.2 mM Na_2_HPO_4_, 26 mM NaHCO_3_, 2 mM CaCl_2_, 1 mM MgCl_2_ and 10 mM dextrose at 305 mOsm. During experiments, slices were submerged and continuously perfused (2-3 ml/min) with aCSF at 32.5°C. All solutions were maintained at pH 7.4 by continuous bubbling with 95% O_2_, 5% CO_2_. Slices were visualized under an upright microscope (Olympus, BX51WI) equipped with Nomarski optics and an electrically insulated 60× water-immersion objective with a long working distance (2 mm) and high numerical aperture (1.0). Recording electrodes were pulled on a horizontal P-1000 pipette puller from borosilicate glass capillaries (Sutter Instrument) with a resistance of 3-5 mΩ.

#### Recordings

The spontaneous firing of sparsely populated SST-INs was recorded in the loose, cell-attached configuration (seal resistance, 50-200 mΩ), voltage-clamp mode, at a command potential at which the amplifier current is at 0 pA with recording electrodes filled with aCSF. Spike amplitude-based identification criteria were systematically applied to evaluate background contamination from nearby spontaneously active interneurons and ensure that the recorded firing reflects activity from the targeted SST-INs. No synaptic blockers were present. Signals were digitized at 100 kHz and logged onto a personal computer with Clampex 10.7 software (Molecular Devices).

To detect sIPSCs, voltage-clamp recordings were performed using the whole-cell configuration of the patch-clamp technique in SST-INs. SST-INs were held at a pipette voltage of −60 mV using an Axopatch 200B (Molecular Devices). Recording electrodes were filled with a CsCl-based internal recording solution containing 145 mM CsCl, 10 mM HEPES, 5 mM ATP-Mg, 0.2 mM GTP-NA and 10 mM EGTA, adjusted to pH 7.2 with CsOH.

Access resistance was monitored during the recordings, and experiments with >20% change were discarded. sIPSC recordings were performed in the presence of the AMPA receptor blocker, 6,7-dinitroquinoxaline-2,3-dione (DNQX; 10 µM). Stock solutions of SR-95531 (Sigma-Aldrich, 5.05986) and DNQX (Sigma-Aldrich, 5.05026) were prepared in water and were diluted to the desired concentration in aCSF and applied locally through a perfusion system for optimal solution exchange in brain slices. Currents were filtered at 2 kHz with a low-pass Bessel filter and digitized at 5-10 kHz using a personal computer equipped with a Digidata 1550A data acquisition board and pCLAMP10.7 software (both from Molecular Devices). Off-line data analysis, curve fitting and figure preparation were performed with Clampfit 10.7 software (Molecular Devices).

#### Data analysis

SST-IN firing was calculated as instantaneous firing rate of the first 100 spikes, which is calculated as the reciprocal of the interspike intervals and refers to the rate of neuronal firing at a specific moment in time. This provides a dynamic, moment-by-moment picture of a neuron's firing behavior. Because this analytical approach is not well suited to capturing burst structure, a small and equally distributed fraction across genotypes of bursting SST-INs (<2%) were excluded from the firing rate analysis. sIPSCs were identified in stable 60 s epochs using the semiautomated template-based detection in Clampfit 10.7 software and were visually confirmed. All detected events were used for event frequency (inter-event interval) and amplitude analysis and represented as cumulative distributions.

### Behavior

All behavioral studies were conducted in the UAB Behavior Assessment Core.

#### Rotarod

Mice used for rotarod were placed in the room 1 h before studies began. These studies were completed during the light phase of the light cycle. Mice were trained at 2 months of age at 4 rpm fixed speed on the San Diego Instruments rotarod device. Three training trials were conducted per day for 2 days at 2-3 months of age. The 2-month experimental studies were subsequently conducted using three trials per day for 3 days post-training at an accelerating speed of 4-40 rpm. At 6 and 12 months of age, mice were re-acclimated to the device by conducting a 5-min training trial on day 1 of experiments. Subsequently, three trials were conducted each day for 3 days over a 5-min period. Times over the three trials were averaged for each mouse at each time point tested, and these data were plotted.

#### Open field

Mice were placed in the room at least 1 h prior to open field testing. Open field testing was completed during the dark phase of the light cycle. Mice were tested using a Coulbourn Instruments open field arena. Briefly, the arena uses infrared technology to determine the position of a mouse at a given time. For a floor plane move to be counted, the animal must pause for at least 1 s between movements. The mice were tested for a 15-min period. Spreadsheets were exported at the end of the trial, and data were compiled. Open field studies were completed at 2-3 months (2 months), 6-7 (6 months) and 12-13 months (12 months) of age.

#### Light–dark box

Mice were placed in the room at least 1 h prior to light–dark box testing. Light–dark box testing was performed during the dark phase of the light cycle. The light–dark box was made of Plexiglass. It had a clear side (45 cm×21 cm×21 cm) and an opaque side (18 cm×21 cm×21 cm), with an opening for the mouse to freely move between both chambers. Mice were placed in the clear side of the light–dark box at the beginning of the trial. The clear side was illuminated using a 60-watt light bulb. Mice were recorded for a 10-min period. The videos were then watched and scored by a researcher who was unaware of individual genotypes. Time spent in the lighted side was recorded. Only the first 5 min of the video were analyzed. Light-dark box studies were completed at 12-18 months.

### Immunofluorescence

Mice were perfused with ice-cold 0.01 M PBS, with subsequent perfusion using 4% paraformaldehyde (PFA) in 0.01 M PBS. The brains of the mice were removed and post-fixed overnight at 4°C in 4% PFA. Subsequently, brains were cryoprotected in 30% sucrose for ∼48 h. Brains were removed from 30% sucrose, wiped and stored at −80°C until use. Brains were sectioned at 40 µm. Sections were rinsed with 0.01 M PBS and blocked using 3% bovine serum albumin and 2% normal serum in 0.01 M PBS. Sections were incubated with primary antibodies overnight at 4°C. The next day, sections were washed three times with 0.01 M PBS, then incubated for 2 h with the appropriate secondary antibodies. Slides were labeled, and sections were mounted using ProLong™ Diamond Antifade Mountant with DAPI (Fisher Scientific, P36971) and left to cure overnight at room temperature in the dark.

### Fluorescence *in situ* hybridization

RNAscope FISH studies were modified from the manufacturer's original protocol and have been used previously (King et al., 2024). Brains were removed and fresh frozen on dry ice. Conditions were kept nuclease free using RNase Zap (Fisher Scientific, AM9780) and 70% ethanol. Brains were sectioned using a Leica cryostat (CM 1950) at 20 µm. Sections were serially placed onto SuperFrost plus slides after every five sections. This was repeated until there were three sections on each slide. Slides were immediately placed on dry ice after three sections were placed on them. Slides were stored long-term at −80°C until use. When ready for use, slices were removed directly from the −80°C freezer and placed directly in a coppin staining jar with 4% PFA made in 1× PBS at 4°C and pH 7.4. All solutions were made using diethyl pyrocarbonate (DEPC)-treated water, except for the 1× wash buffer, which was made using nuclease-free water (Fisher Scientific, 10977-023). A hydrophobic barrier pen was used to draw around each section and treated with hydrogen peroxide (provided with kit), then washed using 1× PBS. Sections were then treated with protease III (provided with kit) and washed using 1× PBS. Sections were incubated with probes at 40°C in a humidified slide box. Probes used were specific for mouse *Sst* (ACD, 404631-C2), human *HTT* (ACD, 473201) and Cre recombinase (ACD, 312281-C3). Each channel was amplified using channel-specific horseradish peroxidases (HRPs; provided with the kit), labeled with a fluorophore, then blocked using the HRP blocker before amplifying the next channel. Slides were washed in between each step using the 1× wash buffer (50× stock provided with the kit). Slides were allowed to dry in a dark place overnight and coverslipped with ProLong™ Diamond Antifade Mountant with DAPI (Fisher Scientific, P36971). Images were taken with a Nikon Ti2 microscope and C2plus camera.

### Statistical analysis

For mHTT reduction in SST-INs, statistical significance was determined using the Mann–Whitney *U*-test. For rotarod and open field experiments, two-way repeated measure ANCOVA, adjusting for weight and genotype, was employed. Tukey's test was used for post hoc comparisons. *P*<0.05 was considered significant.

For electrophysiological experiments, statistical significance of the cumulative distributions of sIPSCs was determined using the Kolmogorov–Smirnov test. The statistical evaluation of the firing rate was performed using unpaired Wilcoxon signed-rank test. All values are expressed as mean±s.e.m. Figures were prepared using GraphPad Prism. For behavior experiments (Figs 2-4), statistical analyses were completed using R. Statistical analyses for Figs 1 and 5 were completed using GraphPad Prism. All statistical tests used and values can be found in Table S1. All means and s.e.m. values can be found in Table S5.

## Supplementary Material

10.1242/dmm.052513_sup1 Supplementary information

## References

[DMM052513C2] Ahveninen, L. M., Stout, J. C., Georgiou-Karistianis, N., Lorenzetti, V. and Glikmann-Johnston, Y. (2018). Reduced amygdala volumes are related to motor and cognitive signs in Huntington's disease: the IMAGE-HD study. *Neuroimage Clin.* 18, 881-887. 10.1016/j.nicl.2018.03.027 29876272 PMC5988225

[DMM052513C3] Anderson, K. E., van Duijn, E., Craufurd, D., Drazinic, C., Edmondson, M., Goodman, N., van Kammen, D., Loy, C., Priller, J. and Goodman, L. V. V. (2018). Clinical management of neuropsychiatric symptoms of huntington disease: expert-based consensus guidelines on agitation, anxiety, apathy, psychosis and sleep disorders. *J. Huntingtons Dis.* 7, 355-366. 10.3233/JHD-180293 30040737 PMC6294590

[DMM052513C4] Andre, V. M., Cepeda, C., Fisher, Y. E., Huynh, M., Bardakjian, N., Singh, S., Yang, X. W. and Levine, M. S. (2011). Differential electrophysiological changes in striatal output neurons in Huntington's disease. *J. Neurosci.* 31, 1170-1182. 10.1523/JNEUROSCI.3539-10.2011 21273402 PMC3071260

[DMM052513C5] Andrew, S. E., Paul Goldberg, Y., Kremer, B., Telenius, H., Theilmann, J., Adam, S., Starr, E., Squitieri, F., Lin, B., Kalchman, M. A. et al. (1993). The relationship between trinucleotide (CAG) repeat length and clinical features of Huntington's disease. *Nat. Genet.* 4, 398-403. 10.1038/ng0893-398 8401589

[DMM052513C6] Bamford, I. J. and Bamford, N. S. (2019). The Striatum's role in executing rational and irrational economic behaviors. *Neuroscientist* 25, 475-490. 10.1177/1073858418824256 30678530 PMC6656632

[DMM052513C7] Barron, J. C., Hurley, E. P. and Parsons, M. P. (2021). Huntingtin and the synapse. *Front. Cell Neurosci.* 15, 689332. 10.3389/fncel.2021.689332 34211373 PMC8239291

[DMM052513C8] Berardelli, A., Hanley, J. J., Booth, P. A. C. and Bevan, M. D. (1999). Pathophysiology of chorea and bradykinesia in Huntington's disease. *Mov. Disord.* 14, 398-403. 10.1002/1531-8257(199905)14:3<398::AID-MDS1003>3.0.CO;2-F 10348461

[DMM052513C9] Bolam, J. P., Hanley, J. J., Booth, P. A. and Bevan, M. D. (2000). Synaptic organisation of the basal ganglia. *J. Anat.* 196, 527-542. 10.1046/j.1469-7580.2000.19640527.x 10923985 PMC1468095

[DMM052513C10] Cepeda, C., Galvan, L., Holley, S. M., Rao, S. P., André, V. M., Botelho, E. P., Chen, J. Y., Watson, J. B., Deisseroth, K. and Levine, M. S. (2013). Multiple sources of striatal inhibition are differentially affected in Huntington's disease mouse models. *J. Neurosci.* 33, 7393-7406. 10.1523/JNEUROSCI.2137-12.2013 23616545 PMC3686572

[DMM052513C11] DiFiglia, M., Sapp, E., Chase, K., Schwarz, C., Meloni, A., Young, C., Martin, E., Vonsattel, J.-P., Carraway, R., Reeves, S. A. et al. (1995). Huntingtin is a cytoplasmic protein associated with vesicles in human and rat brain neurons. *Neuron* 14, 1075-1081. 10.1016/0896-6273(95)90346-1 7748555

[DMM052513C12] Estupina, C., Pinter, A., Belmar, J., Astier, H. and Arancibia, S. (1996). Variations in hypothalamic somatostatin release and content during the estrous cycle in the rat. Effects of ovariectomy and estrogen supplementation. *Neuroendocrinology* 63, 181-187. 10.1159/000126955 9053783

[DMM052513C13] Favuzzi, E., Deogracias, R., Marques-Smith, A., Maeso, P., Jezequel, J., Exposito-Alonso, D., Balia, M., Kroon, T., Hinojosa, A. J., F. Maraver, E. et al. (2019). Distinct molecular programs regulate synapse specificity in cortical inhibitory circuits. *Science* 363, 413-417. 10.1126/science.aau8977 30679375

[DMM052513C14] Fee, C., Prevot, T. D., Misquitta, K., Knutson, D. E., Li, G., Mondal, P., Cook, J. M., Banasr, M. and Sibille, E. (2021). Behavioral deficits induced by somatostatin-positive GABA neuron silencing are rescued by Alpha 5 GABA-A receptor potentiation. *Int. J. Neuropsychopharmacol.* 24, 505-518. 10.1093/ijnp/pyab002 33438026 PMC8278801

[DMM052513C15] Ferrante, R. J., Kowall, N. W. and Richardson, E. P. Jr. (1991). Proliferative and degenerative changes in striatal spiny neurons in Huntington's disease: a combined study using the section-Golgi method and calbindin D28k immunocytochemistry. *J. Neurosci.* 11, 3877-3887. 10.1523/JNEUROSCI.11-12-03877.1991 1836019 PMC6575286

[DMM052513C16] Figueredo-Cardenas, G., Morello, M., Sancesario, G., Bernardi, G. and Reiner, A. (1996). Colocalization of somatostatin, neuropeptide Y, neuronal nitric oxide synthase and NADPH-diaphorase in striatal interneurons in rats. *Brain Res.* 735, 317-324. 10.1016/0006-8993(96)00801-3 8911672

[DMM052513C17] Freeze, B. S., Kravitz, A. V., Hammack, N., Berke, J. D. and Kreitzer, A. C. (2013). Control of basal ganglia output by direct and indirect pathway projection neurons. *J. Neurosci.* 33, 18531-18539. 10.1523/JNEUROSCI.1278-13.2013 24259575 PMC3834057

[DMM052513C18] Gerfen, C. R. (1988). Synaptic organization of the striatum. *J. Electron. Microsc. Tech.* 10, 265-281. 10.1002/jemt.1060100305 3069970

[DMM052513C19] Gerfen, C. R. and Bolam, J. P. (2016). Chapter 1 - the neuroanatomical organization of the Basal Ganglia. In *Handbook of Behavioral Neuroscience* (ed. H. Steiner and K. Y. Tseng), pp. 3-32. Elsevier.

[DMM052513C67] Ghosh, R. and Tabrizi, S. J. (2018). Clinical features of Huntington's disease. *Adv. Exp. Med. Biol.* 1049, 1-28. 10.1007/978-3-319-71779-1_1 29427096

[DMM052513C68] Girgenti, M. J., Wohleb, E. S., Mehta, S., Ghosal, S., Fogaca, M. V. and Duman, R. S. (2019). Prefrontal cortex interneurons display dynamic sex-specific stress-induced transcriptomes. *Transl. Psychiatry* 9, 292. 10.1038/s41398-019-0642-z 31712551 PMC6848179

[DMM052513C20] Gittis, A. H. and Kreitzer, A. C. (2012). Striatal microcircuitry and movement disorders. *Trends Neurosci.* 35, 557-564. 10.1016/j.tins.2012.06.008 22858522 PMC3432144

[DMM052513C21] Goh, A., Wibawa, P., Loi, S. M., Walterfang, M., Velakoulis, D. and Looi, J. C. L. (2018). Neuropsychiatric manifestations of Huntington's disease. *Australas Psychiatry* 26, 366-375. 10.1177/1039856218791036 30012004

[DMM052513C22] Gray, M., Shirasaki, D. I., Cepeda, C., Andre, V. M., Wilburn, B., Lu, X.-H., Tao, J., Yamazaki, I., Li, S.-H., Sun, Y. E. et al. (2008). Full-length human mutant huntingtin with a stable polyglutamine repeat can elicit progressive and selective neuropathogenesis in BACHD mice. *J. Neurosci.* 28, 6182-6195. 10.1523/JNEUROSCI.0857-08.2008 18550760 PMC2630800

[DMM052513C23] Group, T. H. s. D. C. R. (1993). A novel gene containing a trinucleotide repeat that is expanded and unstable on Huntington's disease chromosomes. *Cell* 72, 971-983. 10.1016/0092-8674(93)90585-E 8458085

[DMM052513C24] Han, I., You, Y. M., Kordower, J. H., Brady, S. T. and Morfini, G. A. (2010). Differential vulnerability of neurons in Huntington's disease: the role of cell type-specific features. *J. Neurochem.* 113, 1073-1091. 10.1111/j.1471-4159.2010.06672.x 20236390 PMC2890032

[DMM052513C25] Hedreen, J. C. and Folstein, S. E. (1995). Early loss of neostriatal striosome neurons in Huntington's disease. *J. Neuropathol. Exp. Neurol.* 54, 105-120. 10.1097/00005072-199501000-00013 7815073

[DMM052513C26] Holley, S. M., Galvan, L., Kamdjou, T., Dong, A., Levine, M. S. and Cepeda, C. (2019a). Major contribution of somatostatin-expressing interneurons and cannabinoid receptors to increased GABA synaptic activity in the striatum of Huntington's disease mice. *Front Synaptic. Neurosci.* 11, 14. 10.3389/fnsyn.2019.00014 31139071 PMC6527892

[DMM052513C27] Holley, S. M., Galvan, L., Kamdjou, T., Cepeda, C. and Levine, M. S. (2019b). Striatal GABAergic interneuron dysfunction in the Q175 mouse model of Huntington's disease. *Eur. J. Neurosci.* 49, 79-93. 10.1111/ejn.14283 30472747 PMC8320683

[DMM052513C28] Jackson, A. D., Cohen, J. L., Phensy, A. J., Chang, E. F., Dawes, H. E. and Sohal, V. S. (2024). Amygdala-hippocampus somatostatin interneuron beta-synchrony underlies a cross-species biomarker of emotional state. *Neuron* 112, 1182-1195.e5. 10.1016/j.neuron.2023.12.017 38266646 PMC10994747

[DMM052513C29] King, A. C., Wood, T. E., Rodriguez, E., Parpura, V. and Gray, M. (2020). Differential effects of SNARE-dependent gliotransmission on behavioral phenotypes in a mouse model of Huntington's disease. *Exp. Neurol.* 330, 113358. 10.1016/j.expneurol.2020.113358 32387649 PMC7313419

[DMM052513C30] King, A. C., Payne, E., Stephens, E., Fowler, J. A., Wood, T. E., Rodriguez, E. and Gray, M. (2024). Modulation of SNARE-dependent exocytosis in astrocytes improves neuropathology in Huntington's disease. *Dis. Model. Mech.* 17, dmm052002. 10.1242/dmm.052002 39526491 PMC11583919

[DMM052513C31] Kosinski, C. M., Cha, J.-H., Young, A. B., Persichetti, F., MacDonald, M., Gusella, J. F., Penney, J. B. and Standaert, D. G. (1997). Huntingtin immunoreactivity in the rat neostriatum: differential accumulation in projection and interneurons. *Exp. Neurol.* 144, 239-247. 10.1006/exnr.1997.6441 9168825

[DMM052513C32] Kosinski, C. M., Cha, J.-H., Young, A. B., Mangiarini, L., Bates, G., Schiefer, J. and Schwarz, M. (1999). Intranuclear inclusions in subtypes of striatal neurons in Huntington's disease transgenic mice. *Neuroreport* 10, 3891-3896. 10.1097/00001756-199912160-00031 10716229

[DMM052513C70] Liguz-Lecznar, M., Dobrzanski, G. and Kossut, M. (2022). Somatostatin and somatostatin-containing interneurons-from plasticity to pathology. *Biomolecules* 12, 312. 10.3390/biom12020312 35204812 PMC8869243

[DMM052513C33] Massa, M. G., Scott, R. L., Cara, A. L., Cortes, L. R., Vander, P. B., Sandoval, N. P., Park, J. W., Ali, S. L., Velez, L. M., Wang, H.-B. et al. (2023). Feeding neurons integrate metabolic and reproductive states in mice. *iScience* 26, 107918. 10.1016/j.isci.2023.107918 37817932 PMC10561062

[DMM052513C34] Matamales, M., Bertran-Gonzalez, J., Salomon, L., Degos, B., Deniau, J.-M., Valjent, E., Hervé, D. and Girault, J.-A. (2009). Striatal medium-sized spiny neurons: identification by nuclear staining and study of neuronal subpopulations in BAC transgenic mice. *PLoS ONE* 4, e4770. 10.1371/journal.pone.0004770 19274089 PMC2651623

[DMM052513C35] Matz, O. C. and Spocter, M. (2022). The effect of Huntington's disease on the basal nuclei: a review. *Cureus* 14, e24473. 10.7759/cureus.24473 35651462 PMC9132741

[DMM052513C36] McColgan, P. and Tabrizi, S. J. (2018). Huntington's disease: a clinical review. *Eur. J. Neurol.* 25, 24-34. 10.1111/ene.13413 28817209

[DMM052513C37] Menalled, L., El-Khodor, B. F., Patry, M., Suárez-Fariñas, M., Orenstein, S. J., Zahasky, B., Leahy, C., Wheeler, V., Yang, X. W., MacDonald, M. et al. (2009). Systematic behavioral evaluation of Huntington's disease transgenic and knock-in mouse models. *Neurobiol. Dis.* 35, 319-336. 10.1016/j.nbd.2009.05.007 19464370 PMC2728344

[DMM052513C38] Molero, A. E., Arteaga-Bracho, E. E., Chen, C. H., Gulinello, M., Winchester, M. L., Pichamoorthy, N., Gokhan, S., Khodakhah, K. and Mehler, M. F. (2016). Selective expression of mutant huntingtin during development recapitulates characteristic features of Huntington's disease. *Proc. Natl. Acad. Sci. USA* 113, 5736-5741. 10.1073/pnas.1603871113 27140644 PMC4878495

[DMM052513C39] Orr, H. T., Chung, M.-, Banfi, S., Kwiatkowski, T. J., Servadio, A., Beaudet, A. L., McCall, A. E., Duvick, L. A., Ranum, L. P. W. and Zoghbi, H. Y. (1993). Expansion of an unstable trinucleotide CAG repeat in spinocerebellar ataxia type 1. *Nat. Genet.* 4, 221-226. 10.1038/ng0793-221 8358429

[DMM052513C72] Pittaluga, A., Roggeri, A., Vallarino, G. and Olivero, G. (2021). Somatostatin, a presynaptic modulator of glutamatergic signal in the central nervous system. *Int. J. Mol. Sci.* 22, 5864. 10.3390/ijms22115864 34070785 PMC8198526

[DMM052513C40] Plotkin, J. L. and Goldberg, J. A. (2019). Thinking outside the box (and arrow): current themes in striatal dysfunction in movement disorders. *Neuroscientist* 25, 359-379. 10.1177/1073858418807887 30379121 PMC6529282

[DMM052513C41] Qian, D., Li, W., Xue, J., Wu, Y., Wang, Z., Shi, T., Li, S., Yang, J., Qiu, S., Wang, S. et al. (2022). A striatal SOM-driven ChAT-iMSN loop generates beta oscillations and produces motor deficits. *Cell Rep.* 40, 111111. 10.1016/j.celrep.2022.111111 35858550

[DMM052513C42] Rallapalle, V., King, A. C. and Gray, M. (2021). BACHD mice recapitulate the striatal parvalbuminergic interneuron loss found in Huntington's disease. *Front. Neuroanat.* 15, 673177. 10.3389/fnana.2021.673177 34108866 PMC8180558

[DMM052513C43] Reiner, A., Shelby, E., Wang, H., DeMarch, Z., Deng, Y., Guley, N. H., Hogg, V., Roxburgh, R., Tippett, L. J., Waldvogel, H. J. et al. (2013). Striatal parvalbuminergic neurons are lost in Huntington's disease: implications for dystonia. *Mov. Disord.* 28, 1691-1699. 10.1002/mds.25624 24014043 PMC3812318

[DMM052513C44] Richfield, E. K., Maguire-Zeiss, K. A., Vonkeman, H. E. and Voorn, P. (1995). Preferential loss of preproenkephalin versus preprotachykinin neurons from the striatum of Huntington's disease patients. *Ann. Neurol.* 38, 852-861. 10.1002/ana.410380605 8526457

[DMM052513C73] Roos, R. A. (2010). Huntington's disease: a clinical review. *Orphanet J. Rare Dis.* 5, 40. 10.1186/1750-1172-5-40 21171977 PMC3022767

[DMM052513C45] Rotariu, S., Zalcman, G., Badreddine, N., Appaix, F., Sarno, S., Bureau, I. and Fino, E. (2025). Somatostatin interneurons select dorsomedial striatal representations of the initial motor learning phase. *Cell Rep.* 44, 115670. 10.1016/j.celrep.2025.115670 40333184

[DMM052513C46] Saudou, F. and Humbert, S. (2016). The biology of Huntingtin. *Neuron* 89, 910-926. 10.1016/j.neuron.2016.02.003 26938440

[DMM052513C47] Saunders, A., Macosko, E. Z., Wysoker, A., Goldman, M., Krienen, F. M., de Rivera, H., Bien, E., Baum, M., Bortolin, L., Wang, S. et al. (2018). Molecular diversity and specializations among the cells of the adult mouse brain. *Cell* 174, 1015-1030.e16. 10.1016/j.cell.2018.07.028 30096299 PMC6447408

[DMM052513C48] Schulte, J. and Littleton, J. T. (2011). The biological function of the Huntingtin protein and its relevance to Huntington's Disease pathology. *Curr. Trends Neurol.* 5, 65-78. 22180703 PMC3237673

[DMM052513C49] Sharp, A. H., Loev, S. J., Schilling, G., Li, S.-H., Li, X.-J., Bao, J., Wagster, M. V., Kotzuk, J. A., Steiner, J. P., Lo, A. et al. (1995). Widespread expression of Huntington's disease gene (IT15) protein product. *Neuron* 14, 1065-1074. 10.1016/0896-6273(95)90345-3 7748554

[DMM052513C69] Shenoy, S. A., Zheng, S., Liu, W., Dai, Y., Liu, Y., Hou, Z., Mori, S., Tang, Y., Cheng, J., Duan, W. and Li, C. (2022). A novel and accurate full-length HTT mouse model for Huntington's disease. *Elife* 11, e70217. 10.7554/eLife.70217 35023827 PMC8758142

[DMM052513C50] Shin, J. Y., Fang, Z.-H., Yu, Z.-X., Wang, C.-E., Li, S.-H. and Li, X.-J. (2005). Expression of mutant huntingtin in glial cells contributes to neuronal excitotoxicity. *J. Cell Biol.* 171, 1001-1012. 10.1083/jcb.200508072 16365166 PMC2171327

[DMM052513C51] Smith, Y., MacMillan, J. C., Cheadle, J. P., Fenton, I., Lazarou, L. P., Davies, P., MacDonald, M. E., Gusella, J. F., Harper, P. S. and Shaw, D. J. (1998). Microcircuitry of the direct and indirect pathways of the basal ganglia. *Neuroscience* 86, 353-387. 10.1016/S0306-4522(98)00004-9 9881853

[DMM052513C52] Snell, R. G., MacMillan, J. C., Cheadle, J. P., Fenton, I., Lazarou, L. P., Davies, P., MacDonald, M. E., Gusella, J. F., Harper, P. S. and Shaw, D. J. (1993). Relationship between trinucleotide repeat expansion and phenotypic variation in Huntington's disease. *Nat. Genet.* 4, 393-397. 10.1038/ng0893-393 8401588

[DMM052513C53] Straub, C., Saulnier, J. L., Bègue, A., Feng, D. D., Huang, K. W. and Sabatini, B. L. (2016). Principles of synaptic organization of Gabaergic interneurons in the striatum. *Neuron* 92, 84-92. 10.1016/j.neuron.2016.09.007 27710792 PMC5074692

[DMM052513C54] Taniguchi, H., He, M., Wu, P., Kim, S., Paik, R., Sugino, K., Kvitsani, D., Fu, Y., Lu, J., Lin, Y. et al. (2011). A resource of Cre driver lines for genetic targeting of GABAergic neurons in Cerebral Cortex. *Neuron* 71, 995-1013. 10.1016/j.neuron.2011.07.026 21943598 PMC3779648

[DMM052513C55] Tepper, J. M. and Bolam, J. P. (2004). Functional diversity and specificity of neostriatal interneurons. *Curr. Opin. Neurobiol.* 14, 685-692. 10.1016/j.conb.2004.10.003 15582369

[DMM052513C56] Tepper, J. M. and Koós, T. (2016). Chapter 8 - GABAergic Interneurons of the Striatum. In *Handbook of Behavioral Neuroscience* (ed. H. Steiner and K. Y. Tseng), pp. 157-178. Elsevier.

[DMM052513C57] Tepper, J. M., Koos, T. and Wilson, C. J. (2004). GABAergic microcircuits in the neostriatum. *Trends Neurosci.* 27, 662-669. 10.1016/j.tins.2004.08.007 15474166

[DMM052513C58] Tepper, J. M., Tecuapetla, F., Koós, T. and Ibáñez-Sandoval, O. (2010). Heterogeneity and diversity of striatal GABAergic interneurons. *Front. Neuroanat.* 4, 150. 10.3389/fnana.2010.00150 21228905 PMC3016690

[DMM052513C59] Tepper, J. M., Koós, T., Ibanez-Sandoval, O., Tecuapetla, F., Faust, T. W. and Assous, M. (2018). Heterogeneity and diversity of striatal GABAergic interneurons: update 2018. *Front. Neuroanat.* 12, 91. 10.3389/fnana.2018.00091 30467465 PMC6235948

[DMM052513C71] The Huntington's Disease Collaborative Research Group (1993). A novel gene containing a trinucleotide repeat that is expanded and unstable on Huntington's disease chromosomes. *Cell* 72, 971-983. 10.1016/0092-8674(93)90585-e 8458085

[DMM052513C60] Tremblay, R., Lee, S. and Rudy, B. (2016). GABAergic interneurons in the neocortex: from cellular properties to circuits. *Neuron* 91, 260-292. 10.1016/j.neuron.2016.06.033 27477017 PMC4980915

[DMM052513C61] Voelkl, K., Schulz-Trieglaff, E. K., Klein, R. and Dudanova, I. (2022). Distinct histological alterations of cortical interneuron types in mouse models of Huntington's disease. *Front. Neurosci.* 16, 1022251. 10.3389/fnins.2022.1022251 36225731 PMC9549412

[DMM052513C62] Vonsattel, J. P., Myers, R., Stevens, T., Ferrante, R., Bird, E. and Richardson, E. P. (1985). Neuropathological classification of Huntington's disease. *J. Neuropathol. Exp. Neurol.* 44, 559-577. 10.1097/00005072-198511000-00003 2932539

[DMM052513C63] Wang, N., Gray, M., Lu, X.-H., Cantle, J. P., Holley, S. M., Greiner, E., Gu, X., Shirasaki, D., Cepeda, C., Li, Y. et al. (2014). Neuronal targets for reducing mutant huntingtin expression to ameliorate disease in a mouse model of Huntington's disease. *Nat. Med.* 20, 536-541. 10.1038/nm.3514 24784230 PMC4067603

[DMM052513C64] West, M. J., Ostergaard, K., Andreassen, O. A. and Finsen, B. (1996). Estimation of the number of somatostatin neurons in the striatum: an in situ hybridization study using the optical fractionator method. *J. Comp. Neurol.* 370, 11-22. 10.1002/(SICI)1096-9861(19960617)370:1<11::AID-CNE2>3.0.CO;2-O 8797153

[DMM052513C65] Wibawa, P., Walterfang, M., Malpas, C. B., Glikmann-Johnston, Y., Poudel, G., Razi, A., Hannan, A. J., Velakoulis, D. and Georgiou-Karistianis, N. (2023). Selective perforant-pathway atrophy in Huntington disease: MRI analysis of hippocampal subfields. *Eur. J. Neurol.* 30, 2650-2660. 10.1111/ene.15918 37306313 PMC10946817

[DMM052513C66] Wood, T. E., Barry, J., Yang, Z., Cepeda, C., Levine, M. S. and Gray, M. (2019). Mutant huntingtin reduction in astrocytes slows disease progression in the BACHD conditional Huntington's disease mouse model. *Hum. Mol. Genet.* 28, 487-500. 10.1093/hmg/ddy363 30312396 PMC6337698

