# Supplementary material for: Mutant huntingtin expression in somatostatin-positive interneurons contributes to neurophysiological and behavioral phenotypes in BACHD mice
Source: Dis Model Mech. 2026 Mar 27;19(3):dmm052513. doi: 10.1242/dmm.052513 (PMC13054931; doi:10.1242/dmm.052513)
Supplement: Supplementary information [file dmm-19-052513-s1.pdf]

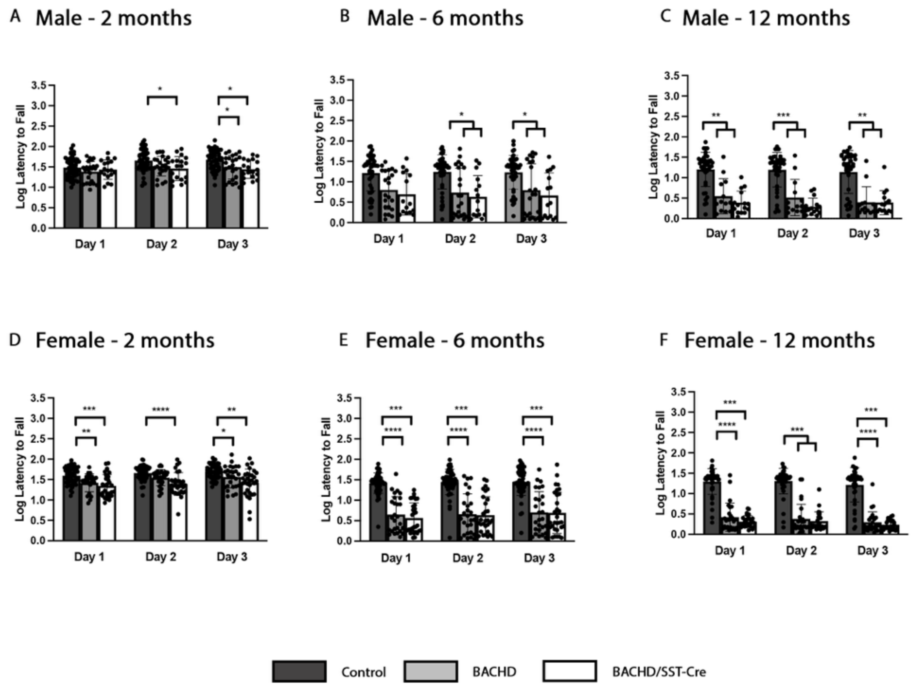

Fig. S1. Reduction of mutant huntingtin in SST-INs does not improve motor coordination in male or female BACHD mice. Rotarod analysis of Control, BACHD, and BACHD/SST-Cre mice at 2, 6 and 12 months of age. (A) Log latency to fall is decreased in both male BACHD and male BACHD/SST-Cre mice relative to control mice, by day 3 at 2 months of age. This trend is observed in male mice at (B) 6 months, and (C) 12 months. (D) Log latency to fall is decreased in both female BACHD and BACHD/SST-Cre mice at 2 months of age by fay 3. This trend also persists in female mice at (E) 6 months, and (F) 12 months. Data was analyzed using a two-way repeated measure ANOVA and adjusted for weight+genotype. Tukey's HSD was used for post hoc comparisons; 2 months n=44-105/genotype, 6 months n=49-100/genotype, 12 months n=41-83/genotype. *p<0.05, ** p<0.01, *** p<0.001, ****p<0.0001.

Disease Models & Mechanisms • Supplementary information

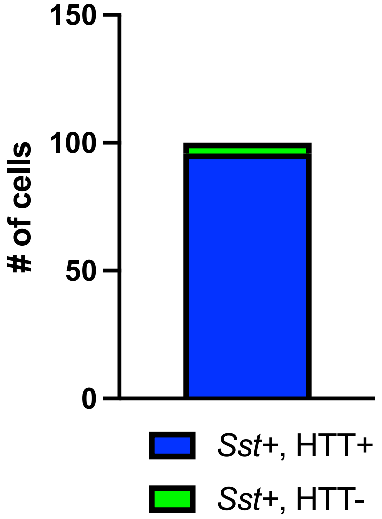

Fig. S2. SST-INs express mutant huntingtin in BACHD mice. Using RNAscope fluorescence *in situ* hybridization, using probes specific to human huntingtin and mouse somatostatin, we found that ~95% of striatal SST-INs express mHTT in BACHD mice. n=3 mice, 48 cells.

Disease Models & Mechanisms • Supplementary information

Table S1. Statistical Tests and Values

Data Analyzed	Test Used	Pairwise Comparisons	Test values
mHTT SST+ cell knockdown	Mann-Whitney U-test	BACHD vs BACHD/SST-Cre	**Mann Whitney U=516.5, p<0.0001**
Rotarod - 2 months -Day 1	Two-way repeated measure ANCOVA, adjusting for weight and genotype interactions w/Tukey's HSD for post hoc comparisons		**Weight Effect: $F(1,125)=7.17 \times 10^{-3}$, p=0.933**
			**Genotype Effect: $F(2,124)=6.63$, $p=2.54\times 10^{-5}$**
		Control vs BACHD	p=0.0029
		Control vs BACHD/SST-Cre	p=0.0005
		BACHD vs BACHD/SST-Cre	p=0.8007
Rotarod - 2 months -Day 2			
		Control vs BACHD	p=0.0203
		Control vs BACHD/SST-Cre	p<0.0001
		BACHD vs BACHD/SST-Cre	p=0.1194
Rotarod - 2 months -Day 3			
		Control vs BACHD	p=0.0054
		Control vs BACHD/SST-Cre	p<0.0001
		BACHD vs BACHD/SST-Cre	p=0.3617
Rotarod - 6 months -Day 1	Two-way repeated measure ANCOVA, adjusting for weight and genotype interactions w/Tukey's HSD for post hoc comparisons		**Weight Effect: $F(1,125)=2.33$, $p=7.96 \times 10^{-4}$**
			**Genotype Effect: $F(2,124)=49.40$, $p=1.66\times 10^{-16}$**
		Control vs BACHD	p=0.0001
		Control vs BACHD/SST-Cre	p<0.0001
		BACHD vs BACHD/SST-Cre	p=0.5745
Rotarod - 6 months -Day 2			
		Control vs BACHD	p<0.0001
		Control vs BACHD/SST-Cre	p<0.0001
		BACHD vs BACHD/SST-Cre	p=0.8901

Rotarod - 6 months -Day 3			
		Control vs BACHD	p=0.0001
		Control vs BACHD/SST-Cre	p<0.0001
		BACHD vs BACHD/SST-Cre	p=0.4907
Rotarod - 12 months -Day 1	Two-way repeated measure ANCOVA, adjusting for weight and genotype interactions w/Tukey's HSD for post hoc comparisons		**Weight Effect: F(1,125)=3.86, p=0.933, p=1.01× 10^{-7}**
			**Genotype Effect: F(2,124)=107.142, p=9.47× 10^{-28}**
		Control vs BACHD	p<0.0001
		Control vs BACHD/SST-Cre	p<0.0001
		BACHD vs BACHD/SST-Cre	p=0.8357
Rotarod - 12 months -Day 2			
		Control vs BACHD	p<0.0001
		Control vs BACHD/SST-Cre	p<0.0001
		BACHD vs BACHD/SST-Cre	p=0.9576
Rotarod - 12 months -Day 3			
		Control vs BACHD	p<0.0001
		Control vs BACHD/SST-Cre	p<0.0001
		BACHD vs BACHD/SST-Cre	p=0.9240
Males - Rotarod - 2 months -Day 1	Two-way repeated measure ANCOVA, adjusting for weight and genotype interactions w/Tukey's HSD for post hoc comparisons		**Weight Effect: F(1,125)=7.17 x 10^{-3}, p=0.933**
			**Genotype Effect: F(2,124)=6.63, p=2.54× 10^{-5}**
		Control vs BACHD	p=0.0782
		Control vs BACHD/SST-Cre	p=0.2247
		BACHD vs BACHD/SST-Cre	p=0.9376
Males - Rotarod - 2 months -Day 2			
		Control vs BACHD	p=0.1184
		Control vs BACHD/SST-Cre	p=0.0128
		BACHD vs BACHD/SST-Cre	p=0.6555

Males - Rotarod - 2 months -Day 3			
		Control vs BACHD	p=0.0460
		Control vs BACHD/SST-Cre	p=0.0349
		BACHD vs BACHD/SST-Cre	p=0.9743
Males - Rotarod - 6 months -Day 1	Two-way repeated measure ANCOVA, adjusting for weight and genotype interactions w/Tukey's HSD for post hoc comparisons		**Weight Effect: F(1,50)=0.741, p=0.765**
			**Genotype Effect: F(2,49)=10.51, p=1.58× 10^{-4}**
		Control vs BACHD	p=0.1676
		Control vs BACHD/SST-Cre	p=0.0594
		BACHD vs BACHD/SST-Cre	p=0.8903
Males - Rotarod - 6 months -Day 2			
		Control vs BACHD	p=0.0486
		Control vs BACHD/SST-Cre	p=0.0271
		BACHD vs BACHD/SST-Cre	p=0.9765
Males - Rotarod - 6 months -Day 3			
		Control vs BACHD	p=0.02754
		Control vs BACHD/SST-Cre	p=0.0315
		BACHD vs BACHD/SST-Cre	p=0.5836
Males - Rotarod - 12 months -Day 1	Two-way repeated measure ANCOVA, adjusting for weight and genotype interactions w/Tukey's HSD for post hoc comparisons		**Weight Effect: F(1,50)= 1.40, p=0.197**
			**Genotype Effect: F(2,49)=20.37, p=3.64 x 10^{-7}**
		Control vs BACHD	p=0.0019
		Control vs BACHD/SST-Cre	p=0.0021
		BACHD vs BACHD/SST-Cre	p=0.9905
Males - Rotarod - 12 months -Day 2			
		Control vs BACHD	p=0.0005
		Control vs BACHD/SST-Cre	p=0.0003
		BACHD vs BACHD/SST-Cre	p=0.9370

Disease Models & Mechanisms • Supplementary information

Males - Rotarod - 12 months -Day 3			
		Control vs BACHD	p=0.0002
		Control vs BACHD/SST-Cre	p=0.0028
		BACHD vs BACHD/SST-Cre	p=0.7830
Females - Rotarod - 2 months -Day 1	Two-way repeated measure ANCOVA, adjusting for weight and genotype interactions w/Tukey's HSD for post hoc comparisons		**Weight Effect: F(1,73)=1.80, p=1.82 X 10^{-1}**
			**Genotype Effect: F(2,72)=10.83, p=7.71× 10^{-5}**
		Control vs BACHD	p=0.0083
		Control vs BACHD/SST-Cre	p=0.0001
		BACHD vs BACHD/SST-Cre	p=0.2863
Females - Rotarod - 2 months -Day 2			
		Control vs BACHD	p=0.0837
		Control vs BACHD/SST-Cre	p=0.0002
		BACHD vs BACHD/SST-Cre	p=0.0625
Females - Rotarod - 2 months -Day 3			
		Control vs BACHD	p=0.0367
		Control vs BACHD/SST-Cre	p=0.0001
		BACHD vs BACHD/SST-Cre	p=0.1145
Females - Rotarod - 6 months -Day 1	Two-way repeated measure ANCOVA, adjusting for weight and genotype interactions w/Tukey's HSD for post hoc comparisons		**Weight Effect: F(1,73)=2.75, p=1.13 X 10^{-3}**
			**Genotype Effect: F(2,72)=48.26, p=5.05× 10^{-14}**
		Control vs BACHD	p=0.0006
		Control vs BACHD/SST-Cre	p<0.0001
		BACHD vs BACHD/SST-Cre	p=0.1881
Females - Rotarod - 6 months -Day 2			
		Control vs BACHD	p=0.0002
		Control vs BACHD/SST-Cre	p<0.0001
		BACHD vs BACHD/SST-Cre	p=0.3981

Disease Models & Mechanisms • Supplementary information

Females - Rotarod - 6 months -Day 3			
		Control vs BACHD	p=0.0003
		Control vs BACHD/SST-Cre	p<0.0001
		BACHD vs BACHD/SST-Cre	p=0.3397
Females - Rotarod - 12 months -Day 1	Two-way repeated measure ANCOVA, adjusting for weight and genotype interactions w/Tukey's HSD for post hoc comparisons		**Weight Effect: F(1,73)=10.24, p=8.7 X 10^{-24}**
			**Genotype Effect: F(2,72)=48.26, p=5.05× 10^{-14}**
		Control vs BACHD	p=0.0003
		Control vs BACHD/SST-Cre	p<0.0001
		BACHD vs BACHD/SST-Cre	p=0.8347
Females - Rotarod - 12 months -Day 2			
		Control vs BACHD	p=0.0001
		Control vs BACHD/SST-Cre	p=0.0001
		BACHD vs BACHD/SST-Cre	p=0.9706
Females - Rotarod - 12 months -Day 3			
		Control vs BACHD	p=0.0001
		Control vs BACHD/SST-Cre	p<0.0001
		BACHD vs BACHD/SST-Cre	p=0.9995
Open Field Floor Plane Velocity - All - 2 months	Two-way repeated measure ANCOVA, adjusting for weight and genotype interactions w/Tukey's HSD for post hoc comparisons		**Weight Effect: F(1,236)=1.493, p=2.99× 10^{-2}**
			**Genotype Effect: F(2,237)=6.63, p=1.580 × 10^{-3}**
		Control vs BACHD	p=0.6870
		Control vs BACHD/SST-Cre	p=0.9794
		BACHD vs BACHD/SST-Cre	p=0.6557
Open Field Floor Plane Velocity - All - 6 months		Control vs BACHD	p=0.0567
		Control vs BACHD/SST-Cre	p=0.0006
		BACHD vs BACHD/SST-Cre	p=0.4274
		Control vs BACHD	p=0.0123

Open Field Floor Plane Velocity - All - 12 months	Control vs BACHD/SST-Cre	p=0.0008
	BACHD vs BACHD/SST-Cre	p=0.7400
Open Field Floor Plane Velocity - Female - 2 months		**Weight Effect: $F(1,123)=1.03$, $p=4.31 \times 10^{-1}$**
		**Genotype Effect: $F(2,124)=9.27$, $p=1.76 \times 10^{-4}$**
	Control vs BACHD	p=0.1969
	Control vs BACHD/SST-Cre	p=0.8962
	BACHD vs BACHD/SST-Cre	p=0.4580
Open Field Floor Plane Velocity - Female - 6 months	Control vs BACHD	p=0.0110
	Control vs BACHD/SST-Cre	p=0.0003
	BACHD vs BACHD/SST-Cre	p=0.5424
Open Field Floor Plane Velocity - Female - 12 months	Control vs BACHD	p=0.0019
	Control vs BACHD/SST-Cre	p=0.0005
	BACHD vs BACHD/SST-Cre	p=0.9173
Open Field Floor Plane Velocity - Male - 2 months		**Weight Effect: $F(1,110)=1.71$, $p=1.33 \times 10^{-2}$**
		**Genotype Effect: $F(2,110)=0.375$, $p=0.0688$**
	Control vs BACHD	p=0.8878
	Control vs BACHD/SST-Cre	p=0.9944
	BACHD vs BACHD/SST-Cre	p=0.9540
Open Field Floor Plane Velocity - Male - 6 months	Control vs BACHD	p=0.6728
	Control vs BACHD/SST-Cre	p=0.1585
	BACHD vs BACHD/SST-Cre	p=0.7428
Open Field Floor Plane Velocity - Male - 12 months	Control vs BACHD	p=0.9470
	Control vs BACHD/SST-Cre	p=0.5308

Disease Models & Mechanisms • Supplementary information

		BACHD vs BACHD/SST-Cre	p=0.8375
Open Field Floor Plane Moves - Male - 2 months	Two-way repeated measure ANCOVA, adjusting for weight and genotype interactions w/Tukey's HSD for post hoc comparisons		**Weight Effect: F(1,110)=1.21, p=2.04 × 10^{-1}**
			**Genotype Effect: F(2,110)=4.96, p=8.681015 × 10^{-3}**
		Control vs BACHD	p=0.0019
		Control vs BACHD/SST-Cre	p=0.0083
		BACHD vs BACHD/SST-Cre	p=0.9548
Open Field Floor Plane Moves - Male - 6 months		Control vs BACHD	p=0.0374
		Control vs BACHD/SST-Cre	p=0.6650
		BACHD vs BACHD/SST-Cre	p=0.4910
Open Field Floor Plane Moves - Male - 12 months		Control vs BACHD	p=0.2646
		Control vs BACHD/SST-Cre	p=0.7747
		BACHD vs BACHD/SST-Cre	p=0.6524
Open Field Floor Plane Moves - Female - 2 months			**Weight Effect: F(1,124)= 2.08, p=5.27 x10^{-4}**
			**Genotype Effect: F(2,124)=9.20, p=1.87951 × 10^{-4}**
		Control vs BACHD	p=0.1829
		Control vs BACHD/SST-Cre	p=0.1339
		BACHD vs BACHD/SST-Cre	p=0.9700
Open Field Floor Plane Moves - Female - 6 months		Control vs BACHD	p=0.0581
		Control vs BACHD/SST-Cre	p=0.0637
		BACHD vs BACHD/SST-Cre	p=0.9743
Open Field Floor Plane Moves - Female - 12 months		Control vs BACHD	p=0.5754
		Control vs BACHD/SST-Cre	p=0.7207
		BACHD vs BACHD/SST-Cre	p=0.9755
Open Field Floor Plane Moves - All - 2 months			**Weight Effect: F(1,237)=2.60, P=6.42 x 10^{-7}**
			**Genotype Effect: F(2,237)=12.66, p=5.979752 × 10^{-6}**
		Control vs BACHD	p=0.0216

Disease Models & Mechanisms • Supplementary information

		Control vs BACHD/SST-Cre	p=0.0935
		BACHD vs BACHD/SST-Cre	p=0.9047
Open Field Floor Plane Moves - All - 6 months		Control vs BACHD	p=0.0079
		Control vs BACHD/SST-Cre	p=0.0688
		BACHD vs BACHD/SST-Cre	p=0.8024
Open Field Floor Plane Moves - All - 12 months		Control vs BACHD	p=0.6124
		Control vs BACHD/SST-Cre	p=0.9788
		BACHD vs BACHD/SST-Cre	p=0.7132
Light-Dark Box - All	One-way ANOVA w/ Tukey's HSD for post hoc comparisons		**Weight Effect: F(2,97) = 2.229, p=0.139**
			**Genotype Effect: F(2,97)=4.29, p=0.0164**
		Control vs BACHD	p=0.0126
		Control vs BACHD/SST-Cre	p=0.3778
		BACHD vs BACHD/SST-Cre	p=0.3682
Light-Dark Box - Female	One-way ANOVA w/ Tukey's HSD for post hoc comparisons		**Weight Effect: F(2,56)=1.39, p=0.412**
			**Genotype Effect: F(2,56)=1.39, p=0.2581**
		Control vs BACHD	p=0.2983
		Control vs BACHD/SST-Cre	p=0.4191
		BACHD vs BACHD/SST-Cre	p=0.9640
Light-Dark Box - Male	One-way ANOVA w/ Tukey's HSD for post hoc comparisons		**Weight Effect: F(2,38)=4.79, p=0.165**
			**Genotype Effect: F(2,38)=4.79, p=0.0139**
		Control vs BACHD	p=0.0148
		Control vs BACHD/SST-Cre	p=0.9739
		BACHD vs BACHD/SST-Cre	p=0.0478
SST-IN spontaneous firing	One-way ANOVA w/ Tukey's HSD for post hoc comparisons		**Genotype Effect: F(2,15)=11.20, p=0.0011**
		Control vs BACHD	p=0.0015
		Control vs BACHD/SST-Cre	p=0.9468

		BACHD vs BACHD/SST-Cre	p=0.0035
SST-IN sIPSC frequency	Kolmogorov-Smirnov test	Control vs BACHD	p=0.4999
		Control vs BACHD/SST-Cre	p<0.0001
		BACHD vs BACHD/SST-Cre	p<0.0001
SST-IN sIPSC amplitude	Kolmogorov-Smirnov test	Control vs BACHD	p<0.0001
		Control vs BACHD/SST-Cre	p=0.0159
		BACHD vs BACHD/SST-Cre	p<0.0001

Table S2. Weight (grams) of Animals Used in Behavioral Tests, ± SEM

	**2 months**	**6 months**	**12 months**
**Control**	24.45 ± 0.39	31.14 ± 0.57	37.07 ± 0.82
**BACHD/WT**	27.39 ± 0.57	43.48 ± 0.97	55.13 ± 1.01
**BACHD/SST-Cre**	28.48 ± 0.67	44.57 ± 1.01	55.31 ± 1.29

Disease Models & Mechanisms • Supplementary information

Table S3. Samples Sizes for Behavioral Experiments

**Rotarod**						
	**2 months**		**6 months**		**12 months**	
	**Male**	**Female**	**Male**	**Female**	**Male**	**Female**
**Control (WT/WT+ SST-Cre/WT)**	52	53	48	51	40	42
**BACHD**	23	27	22	27	13	26
**BACHD/SST-Cre**	18	30	15	30	15	26
**Open Field**						
	**2 months**		**6 months**		**12 months**	
	**Male**	**Female**	**Male**	**Female**	**Male**	**Female**
**Control (WT/WT+ SST-Cre/WT)**	55	52	45	42	31	40
**BACHD**	27	27	23	26	9	24
**BACHD/SST-Cre**	23	27	14	27	12	26
**Light Dark Box**						
					**12 months**	
					**Male**	**Female**
**Control (WT/WT+ SST-Cre/WT)**					31	40
**BACHD**					9	24
**BACHD/SST-Cre**					12	26

Disease Models & Mechanisms • Supplementary information

Table S4. Sample Sizes for Electrophysiological Experiments

Spontaneous Firing		
	12 months	
	Male	Female
**Control (WT/WT+ SST-Cre/WT)**	6 mice (3 WT/WT+ 3 SST-Cre/WT)<br>25 cells	1 mouse (WT)<br>2 cells
**BACHD**	1 mouse<br>8 cells	4 mice<br>22 cells
**BACHD/SST-Cre**	2 mice<br>12 cells	4 mice<br>24 cells

sIPSC		
	12 months	
	Male	Female
**Control (WT/WT+ SST-Cre/WT)**	5 mice	2 mice
**BACHD**	4 mice	1 mouse
**BACHD/SST-Cre**	1	5

Table S5. Means ± SEM for Behavioral Experiments

Rotarod – Mean Latency to Fall, in seconds, ± SEM			
	**2 months**	**6 months**	**12 months**
**Control (WT/WT+ SST-Cre/WT)**	48.45 ± 1.91	36.8 ± 2.19	32.75 ± 2.89
**BACHD**	28.77 ± 1.74	10.65 ± 1.74	8.14 ± 1.42
**BACHD/SST-Cre**	22.66 ± 1.30	4.2 ± 0.96	2.42 ± 0.22
**Open Field – Mean Floor Plane Velocity, in centimeters per second, ± SEM**			
	**2 months**	**6 months**	**12 months**
**Control (WT/WT+ SST-Cre/WT)**	5.44 ± 0.11	5.92 ± 0.18	6.01 ± 0.22
**BACHD**	5.709 ± 0.14	5.38 ± 0.16	4.78 ± 0.25
**BACHD/SST-Cre**	5.55 ± 0.11	4.93 ± 0.21	4.46 ± 0.18
**Open Field – Mean Floor Plane Moves, ± SEM**			
	**2 months**	**6 months**	**12 months**
**Control (WT/WT+ SST-Cre/WT)**	131.37 ± 1.69	117.51 ± 1.76	110.08 ± 2.44
**BACHD**	120.52 ± 2.26	106.82 ± 2.41	107.97 ± 2.56
**BACHD/SST-Cre**	122.06 ± 2.00	108.12 ± 2.42	111.60 ± 2.25
**Light-Dark Box – Mean Time in Light, in seconds ± SEM**			
			**12 months**
**Control (WT/WT+ SST-Cre/WT)**			125.96 ± 7.78
**BACHD**			87.16 ± 9.25
**BACHD/SST-Cre**			108.08 ± 12.25
